# On the Lift, Related Privacy Measures, and Applications to Privacy–Utility Trade-Offs †

**DOI:** 10.3390/e25040679

**Published:** 2023-04-18

**Authors:** Mohammad Amin Zarrabian, Ni Ding, Parastoo Sadeghi

**Affiliations:** 1 College of Engineering, Computing and Cybernetics, Australian National University, Canberra, ACT 2601, Australia; 2 School of Computing and Information Systems, University of Melbourne, Parkville, VIC 3010, Australia; ni.ding@unimelb.edu.au; 3 School of Engineering and Information Technology, University of New South Wales, Canberra, ACT 2600, Australia

**Keywords:** local information privacy, local differential privacy, watchdog privacy mechanism, optimal random response

## Abstract

This paper investigates lift, the likelihood ratio between the posterior and prior belief about sensitive features in a dataset. Maximum and minimum lifts over sensitive features quantify the adversary’s knowledge gain and should be bounded to protect privacy. We demonstrate that max- and min-lifts have a distinct range of values and probability of appearance in the dataset, referred to as *lift asymmetry*. We propose asymmetric local information privacy (ALIP) as a compatible privacy notion with lift asymmetry, where different bounds can be applied to min- and max-lifts. We use ALIP in the watchdog and optimal random response (ORR) mechanisms, the main methods to achieve lift-based privacy. It is shown that ALIP enhances utility in these methods compared to existing local information privacy, which ensures the same (symmetric) bounds on both max- and min-lifts. We propose subset merging for the watchdog mechanism to improve data utility and subset random response for the ORR to reduce complexity. We then investigate the related lift-based measures, including $\ell_{1}$-norm, $\chi^{2}$-privacy criterion, and α-lift. We reveal that they can only restrict max-lift, resulting in significant min-lift leakage. To overcome this problem, we propose corresponding lift-inverse measures to restrict the min-lift. We apply these lift-based and lift-inverse measures in the watchdog mechanism. We show that they can be considered as relaxations of ALIP, where a higher utility can be achieved by bounding only average max- and min-lifts.

## 1. Introduction

With the recent emergence of “Big-Data”, generating, sharing, and analysing data are proliferating via the advancement of communication systems and machine learning methods. While sharing datasets is essential to achieve social and economic benefits, it may lead to the leakage of private information, which has raised great concern about the privacy preservation of individuals. The main approach to protect privacy is perturbing the data via a privacy mechanism. Consider some raw data denoted by random variable *X* and some sensitive features denoted by *S*, which are correlated via a joint distribution $P_{SX}\neq P_{S}\times P_{X}$. A privacy mechanism (characterised by the transition probability $P_{Y|X}$) is applied to publish *Y* as a sanitised version of *X* to protect *S*.

The design of a privacy mechanism depends on the privacy measure. Differential privacy (DP) [1,2,3] is a widely used notion of privacy. DP restricts the chance of revealing the individual’s presence in a dataset from the outcome of analysis over that dataset [4]. It ensures that neighboured sensitive features *s* and $s^{\prime }$, which differ in only one entry, result in a similar output probability distribution, by restricting the ratio between posterior beliefs $P_{Y|S}\left(y|s\right)/P_{Y|S}\left(y|s^{\prime }\right)$ below a threshold $e^{\varepsilon }.$ The neighbourhood assumption is relaxed in the local differential privacy (LDP) [5,6,7,8,9], where the ratio between posterior beliefs is restricted below $e^{\varepsilon }$ for *any* two sensitive features *s* and $s^{\prime }$, denoted by ε-LDP. The quantity of ε is known as the *privacy budget*. DP and LDP are considered context-free privacy notions, i.e., they do not take into account the prior distribution $P_{S}$. In contrast, in information-theoretic (IT) privacy, also known as context-aware privacy [10,11], it is assumed that the distribution of data or an estimation of them is available. Some of the dominant IT privacy measures are mutual information (MI) [10,12,13], maximal leakage [14,15,16], α-leakage [17], and local information privacy (LIP) [11,18,19,20,21,22,23,24,25,26,27,28]. A challenge is that while data perturbation restricts privacy leakage, it necessarily reduces data resolution and datasets’ usefulness. Therefore, a privacy mechanism is desired to deliver a satisfactory level of data utility. Depending on the application, data utility is quantified either by measures of similarity between *X* and *Y*, such as f-divergence [7] and MI [7,12], or measures of dissimilarity and error, such as Hamming distortion [8,9] and mean square error [22], respectively. This tension between privacy and utility is known as the privacy–utility trade-off (PUT).

In this paper, we consider lift, a pivotal element in IT privacy measures, which is the likelihood ratio between the posterior belief $P_{S|Y}\left(s|y\right)$ and prior belief $P_{S}\left(s\right)$ about sensitive features in a dataset:

(1) $$l\left(s,y\right)=\frac{P_{S|Y}\left(s|y\right)}{P_{S}\left(s\right)}=\frac{P_{SY}\left(s,y\right)}{P_{S}\left(s\right)P_{Y}\left(y\right)}.$$

The logarithm of the lift i(s,y)=logl(s,y), which we call *log-lift*, is the information density [24]. For each *y*, the more $P_{S|Y}\left(s|y\right)$ differs from $P_{S}\left(s\right)$, the more the adversary gains knowledge about *s* [29]. Consequently, both min-lift and max-lift, denoted by $\min_{s}l\left(s,y\right)$ and $\max_{s}l\left(s,y\right)$, respectively, quantify the highest privacy leakage for each *y*. In [29], the role of min-lift and max-lift in privacy breach was proposed, based on which information privacy was introduced in [18]. Accordingly, min-lift is associated with revealing what values are less probable for *s* after observing *y*, while max-lift is associated with the more probable values. In addition, recently, other operational meanings for max-lift have been revealed in guessing frameworks [30] and quantitative information flow [31]. In LIP, min-lift and max-lift are bounded below and above by thresholds $e^{-\varepsilon }$ and $e^{\varepsilon }$, respectively, to restrict the adversary’s knowledge gain, denoted by ε-LIP. The main privacy mechanisms to achieve ε-LIP are the watchdog mechanism [24,25] and optimal random response (ORR) [28]. Watchdog mechanism bipartitions the alphabet of *X* into low-risk and high-risk symbols, and only high-risk ones are randomised. It was proved in [25] that *X*-invariant randomisation (e.g., merging all high-risk symbols) minimises privacy leakage for the watchdog mechanism. ORR is an optimal mechanism for ε-LIP, which maximises MI as the utility measure.

### Contributions

We investigate lift and its related privacy notions such as LIP. We demonstrate that min-lift and max-lift have distinct values and probability of appearance in the dataset. More specifically, min-lifts have a broader range of values than max-lifts, while max-lifts have a higher likelihood $P_{SY}\left(s,y\right)$ of appearing in the dataset. We call this property *lift asymmetry*. However, ε-LIP allocates symmetric privacy budgets to $\min_{s}i\left(s,y\right)$ and $\max_{s}i\left(s,y\right)$ (−ε and ε, respectively), which is incompatible with the lift asymmetry. Thus, we propose asymmetric-LIP (ALIP) as an amenable privacy notion to the lift properties, where asymmetric privacy budgets can be allocated to $\min_{s}i\left(s,y\right)$ and $\max_{s}i\left(s,y\right)$, denoted by $-\varepsilon_{l}$ and $\varepsilon_{u}$, respectively. We demonstrate that ALIP implies ε-LDP and can result in better utility than LIP in the watchdog and ORR mechanisms. Utility increases by relaxing the bound on the min-lift, which has a lower probability of appearance in the dataset.

We propose two randomization methods to overcome the low utility of the watchdog mechanism and the high complexity of the ORR mechanism. In the watchdog mechanism, *X*-invariant randomisation perturbs all high-risk symbols together and deteriorates data resolution and utility. On the other hand, ORR suffers from high complexity, which is exponential in the size of datasets. To overcome these problems, we propose *subset merging* and *subset random response* (SRR) perturbation methods that make finer subsets of high-risk symbols and privatise each subset separately. Subset merging enhances utility in the watchdog mechanism by applying *X*-invariant randomisation to disjoint subsets of high-risk symbols. In addition, SRR relaxes the complexity of ORR for large datasets by applying random response solutions on disjoint subsets of high-risk symbols, which results in near-optimal utility.

Besides LIP, we also consider some recently proposed privacy measures, which we call *lift-based* measures, including $\ell_{1}$-norm [32], $\chi^{2}$-strong privacy [33], and α-lift [34]. They have been proposed as the privacy notions stronger than their corresponding average leakages: the total variation distance [35], $\chi^{2}$-divergence [36], and Sibson MI [16,34], respectively. We clarify that they only bound max-lift leakage and can cause significant min-lift leakage. Therefore, we propose a corresponding modified version of these measures to restrict min-lift leakage, which we call *lift-inverse* measures. We apply lift-based and lift-inverse measures in the watchdog mechanism with subset merging randomisation to investigate their PUT. They result in higher utility than ALIP since they are functions of average lift over sensitive features and, thus, can be considered as relaxations of the max- and min-lift.

## 2. Preliminaries

### 2.1. Notation

We use the following notation throughout the paper. Capital letters denote discrete random variables, corresponding capital calligraphic letters denote their finite supports, and lowercase letters denote any of their realisations. For example, a random variable *X* has the support X, and its realisation is x∈X. For random variables *S* and *X*, we use $P_{SX}$ to indicate their joint probability distribution, $P_{S|X}$ for the conditional distribution of *S* given *X*, and $P_{S}$ and $P_{X}$ for the marginal distributions. Bold capital and lowercase letters are used for matrices and vectors, respectively, and lowercase letters for the corresponding elements of the vectors, e.g., $v={\left[v_{1},v_{2},\cdots ,v_{n}\right]}^{T}$. We also use |·| for the cardinality of a set, e.g., |X|. We denote the natural logarithm by log and the set of integers {1,2,⋯,n} by [n]. The indicator function is shown by $1_{\left\{f\right\}}$, which is 1 when *f* is true and zero otherwise.

### 2.2. System Model and Privacy Measures

Consider some useful data intended for sharing and denoted by random variable *X* with alphabet X. It is correlated with some sensitive features *S* with the alphabet S through a discrete joint distribution $P_{SX}$. To protect the sensitive features, a privacy mechanism is applied to generate a sanitised version of *X*, denoted by *Y* with the alphabet Y. We assume $P_{S}$ and $P_{X}$ have full support, and $P_{Y|X,S}\left(y|x,s\right)=P_{Y|X}\left(y|x\right)$, which results in the Markov chain S−X−Y.

The main privacy measure is lift (since we assume $P_{S}$ and $P_{Y}$ have full supports, l(s,y) is finite), given in (1). Lift and its logarithm, log-lift, quantify multiplicative information gain on each sensitive feature s∈S via accessing y∈Y. There are two cases: $l\left(s,y\right)>1\Rightarrow P_{S|Y}\left(s|y\right)>P_{S}\left(s\right)$ indicates the increment of the belief about *s* after releasing *y*; $l\left(s,y\right)\leq 1\Rightarrow P_{S|Y}\left(s|y\right)\leq P_{S}\left(s\right)$ means that releasing *y* decreases the belief. The more the posterior belief deviates from the prior belief, the more an adversary gains knowledge about *s*. Thus, for each y∈Y, the $\max_{s}l\left(s,y\right)$ and $\min_{s}l\left(s,y\right)$ determine the highest knowledge gain of sensitive features, and they should be restricted to protect privacy. We use the following notation for these quantities:

(2) $$\Psi \left(y\right)≜\min_{s\in S}l\left(s,y\right)\;\mathrm{and}\;\Lambda \left(y\right)≜\max_{s\in S}l\left(s,y\right).$$

In Appendix A, we explain the operational meaning of Ψ(y) and Λ(y) in privacy breach based on the work in [29].

The lift has been applied in local information privacy [24,25,28] to provide protection of sensitive features, and is defined as follows.

**Definition** **1.** *For $\varepsilon \in R_{+}$, a privacy mechanism M:X→Y is ε-local information private or ε-LIP, with respect to S, if for all y∈Y,*

(3) $$e^{-\varepsilon }\leq \Psi \left(y\right)\;\mathrm{and}\;\Lambda \left(y\right)\leq e^{\varepsilon }.$$

Another instance-wise measure is local differential privacy [5,6,28],

**Definition** **2.** *For $\varepsilon \in R_{+}$, a privacy mechanism M:X→Y is ε-local differential private or ε-LDP, with respect to S, if for all $s,s^{\prime }\in S$ and all y∈Y,*

(4) $$\Gamma \left(y\right)=\underset{s,s^{\prime }\in S}{\sup}\frac{P_{Y|S}\left(y|s\right)}{P_{Y|S}\left(y|s^{\prime }\right)}=\frac{\Lambda (y)}{\Psi (y)}\leq e^{\varepsilon }.$$

## 3. Asymmetric Local Information Privacy

**Figure 1:**
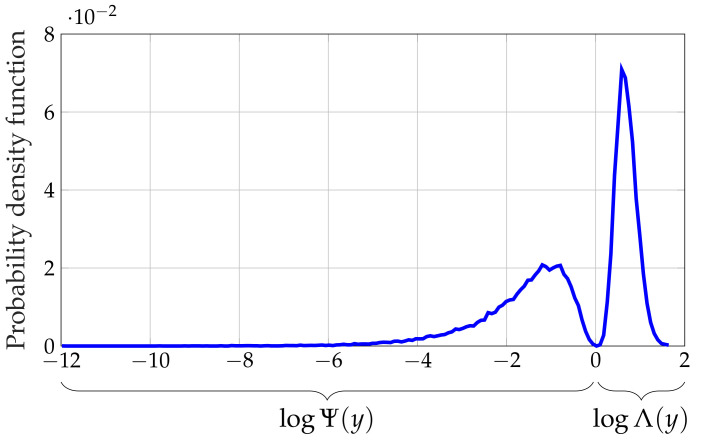
Histogram of $\log\Psi \left(y\right)=\min_{s}i\left(s,y\right)$ and $\log\Lambda \left(y\right)=\max_{s}i\left(s,y\right)$ for $10^{3}$ randomly generated distributions, where |X|=17, |S|=5.

According to (3), LIP restricts the decrement of logΨ(y) and increment of logΛ(y) by the symmetric bounds. However, we demonstrate that these metrics have a distinct range of values and probabilities of appearance in the dataset, $P_{SY}\left(s,y\right)$. We plot the histogram of logΨ(y) and logΛ(y) for $10^{3}$ randomly generated distributions in Figure 1, where |X|=17 and |S|=5. In this figure, the range of logΨ(y) is [−12,−0.06], much larger than the range of logΛ(y), [0.02,1.64]. Moreover, the maximum probability of logΨ(y) is much lower than the maximum probability of logΛ(y). We refer to these properties as *lift asymmetry*. Since high values of |logΨ(y)| have a significantly lower probability (for example, in Figure 1, the probability of |logΨ(y)|≥6 is near zero) than the logΛ(y), we can relax the min-lift privacy by allocating a higher privacy budget to it while applying a stricter bound for the max-lift. Thus, we propose asymmetric local information privacy (ALIP), where we consider different privacy budgets $\varepsilon_{l}$ and $\varepsilon_{u}$ for |logΨ(y)| and logΛ(y), respectively.

This will result in the following notion of privacy, which is more compatible with the lift asymmetry property.

**Definition** **3.** *For $\varepsilon_{l},\varepsilon_{u}\in R_{+}$, a privacy mechanism M:X→Y is $\left(\varepsilon_{l},\varepsilon_{u}\right)$-asymmetric local information private, or $\left(\varepsilon_{l},\varepsilon_{u}\right)$-ALIP, with respect to S, if for all y∈Y,*

(5) $$e^{-\varepsilon_{l}}\leq \Psi \left(y\right)\;\mathrm{and}\;\Lambda \left(y\right)\leq e^{\varepsilon_{u}}.$$

The following proposition indicates how $\left(\varepsilon_{l},\varepsilon_{u}\right)$-ALIP restricts average privacy leakage measures and LDP.

**Proposition** **1.** *If $\left(\varepsilon_{l},\varepsilon_{u}\right)$-ALIP is satisfied, then*

 *1.*  *$I\left(S;Y\right)\leq \varepsilon_{u}$;*
 *2.*  *$T\left(S;Y\right)\leq \frac{1}{2}\left(e^{\varepsilon_{u}}-1\right)$ and $\chi^{2}\left(S;Y\right)\leq {\left(e^{\varepsilon_{u}}-1\right)}^{2}$;*
 *3.*  *$I_{\alpha }^{S}\left(S;Y\right)\leq \frac{\alpha }{\alpha -1}\varepsilon_{u}$ and $I_{\alpha }^{A}\left(S;Y\right)\leq \frac{\alpha }{\alpha -1}\varepsilon_{u}$;*
 *4.*  *ε-LDP is satisfied where $\varepsilon =\varepsilon_{l}+\varepsilon_{u}$;*

*where T(S;Y) is the total variation distance, $\chi^{2}\left(S;Y\right)$ is $\chi^{2}$-divergence, $I_{\alpha }^{S}\left(S;Y\right)$ is Sibson MI, and $I_{\alpha }^{A}\left(S;Y\right)$ is Arimoto MI.*

**Proof.**  The proof is given in Appendix B.    □

Proposition 1-1–3 demonstrate that average measures are bounded with the max-lift privacy budget. In Section 3.1, we show that ALIP can enhance utility via relaxing min-lift $\varepsilon_{l}>\varepsilon_{u}$, where a smaller upper bound is allocated to the max-lift and average measures in Proposition 1. Proposition 1–4 shows the relationship between $\left(\varepsilon_{l},\varepsilon_{u}\right)$-ALIP and ε-LDP. We introduce a variable λ∈(0,1) to have a convenient representation of this relationship as follows: for an LDP privacy budget ε, if we set $\varepsilon_{l}=\lambda \varepsilon$ and $\varepsilon_{u}=\left(1-\lambda \right)\varepsilon$, we have $\varepsilon_{l}+\varepsilon_{u}=\varepsilon$. Thus, varying λ gives rise to different $\left(\varepsilon_{l},\varepsilon_{u}\right)$-ALIP scenarios within the same budget for ε-LDP. If λ<0.5, we have relaxation on the max-lift privacy; if λ>0.5, it implies relaxation on the min-lift privacy. When λ=0.5, we have the *symmetric* case of $\frac{\varepsilon }{2}$-LIP, where $\varepsilon_{l}=\varepsilon_{u}=\frac{\varepsilon }{2}$.

### 3.1. ALIP Privacy–Utility Trade-Off

In this subsection, we propose a watchdog mechanism based on ALIP and LDP and an asymmetric ORR (AORR) mechanism for ALIP to perturb data and achieve privacy protection. We observe the PUT of ALIP and LDP, where the utility is measured by MI between *X* and *Y*, I(X;Y).

#### 3.1.1. Watchdog Mechanism

Watchdog privacy mechanism bipartitions X into low-risk and high-risk subsets denoted by $X_{L}$ and $X_{H}$, respectively, and only randomises high-risk symbols. In the existing LIP, $X_{L}$ and $X_{H}$ are determined by symmetric bounds. We propose to use ALIP to obtain $X_{L}$ and $X_{H}$:

(6) $$\begin{array}{c} X_{L}≜\left\{x\in X:e^{-\varepsilon_{l}}\leq \Psi \left(x\right)\;and\;\Lambda \left(x\right)\leq e^{\varepsilon_{u}}\right\}\;and\;X_{H}=X\setminus X_{L}. \end{array}$$

For LDP, $X_{L}$ and $X_{H}$ are given by

(7) $$\begin{array}{c} X_{L}≜\left\{x\in X:\Gamma \left(x\right)\leq e^{\varepsilon }\right\}\;\;and\;X_{H}=X\setminus X_{L}. \end{array}$$

After obtaining $X_{L}$ and $X_{H}$, the privacy mechanism will be

(8) $$M=\left\{\begin{array}{cc} 1_{\left\{x=y\right\}}, & x,y\in X_{L}=Y_{L},\\ r(y|x), & x\in X_{H},y\in Y_{H},\\ 0, & otherwise, \end{array}\right.$$

where $1_{\left\{x=y\right\}}$ indicates the publication of low-risk symbols without alteration, and r(y|x) is the randomisation on high-risk symbols, where $\sum_{y\in Y_{H}}r\left(y|x\right)=1$.

An instance of r(y|x) is the *X*-invariant randomisation, if r(y|x)=R(y) for $x\in X_{H},y\in Y_{H}$, and $\sum_{y\in Y_{H}}R\left(y\right)=1$. An example of R(y) is the uniform randomisation $R\left(y\right)=\frac{1}{|Y_{H}|}$ with the special case of *complete merging*, where $|Y_{H}|=1$, and all $x\in X_{H}$ are mapped to one super symbol $y^{*}\in Y_{H}$. It was proved in [25] for LIP that *X*-invariant randomisation minimises privacy leakage in $X_{H}$. Accordingly, if we apply ALIP in the watchdog mechanism, for $X_{H}\neq ⌀$, the minimum leakages over $X_{H}$ are

(9) $$\begin{array}{c} {\bar{\varepsilon }}_{u}:=\max_{s\in S}i\left(s,X_{H}\right)=\max_{s\in S}\log l\left(s,X_{H}\right)=\max_{s\in S}\log\frac{P(X_{H}|s)}{P(X_{H})}, \end{array}$$

(10) $$\begin{array}{c} {\bar{\varepsilon }}_{l}:=\left|\min_{s\in S}i\left(s,X_{H}\right)\right|=\left|\min_{s\in S}\log l\left(s,X_{H}\right)\right|=\left|\min_{s\in S}\log\frac{P(X_{H}|s)}{P(X_{H})}\right|, \end{array}$$

where $P\left(X_{H}|s\right)=\sum_{x\in X_{H}}P_{X|S}\left(x|s\right)$ and $P\left(X_{H}\right)=\sum_{x\in X_{H}}P_{X}\left(x\right).$

*X*-invariant randomisation is also applicable for LDP, and the following theorem shows that it minimises LDP privacy leakage in $X_{H}$.

**Theorem** **1.** *In the LDP watchdog mechanism where $X_{L}$ and $X_{H}$ are determined according to *(7)*, X-invariant randomisation minimises privacy leakage in $X_{H}$ measured by Γ(y) in *(4)*.*

**Proof.**  The proof is given in Appendix C.    □

In the watchdog mechanism with *X*-invariant randomisation, the resulting utility measured by MI between *X* and *Y* is given by

(11) $$I\left(X;Y\right)=H\left(X\right)-\sum_{x\in X_{H}}P_{X}\left(x\right)\log\frac{P(X_{H})}{P_{X}\left(x\right)}.$$

In [25], it was verified that I(X;Y) in (11) is monotonic in $X_{H}$: if $X_{H}^{{}^{\prime }}\subset X_{H}$ then $I\left(X;Y\right)<I^{{}^{\prime }}\left(X;Y\right)$, where $I^{{}^{\prime }}\left(X;Y\right)$ is the resulting utility of $X_{H}^{{}^{\prime }}$.

**Proposition** **2.** *In the watchdog mechanism with X-invariant randomisation, for a given LDP privacy budget ε, λ∈(0,1), and ALIP privacy budgets $\varepsilon_{l}=\lambda \varepsilon ,\varepsilon_{u}=\left(1-\lambda \right)\varepsilon$, LDP results in higher utility and than ALIP.*

**Proof.**  Denote the high-risk subset for LDP by $X_{H}^{{}^{\prime }}$ and for ALIP by $X_{H}$. We have

$$X_{H}^{{}^{\prime }}=\left\{x\in X:\frac{\Lambda (x)}{\Psi (x)}>e^{\varepsilon }\right\}\;\mathrm{and}\;X_{H}=\left\{x\in X:\Lambda \left(x\right)>e^{(1-\lambda )\varepsilon }\;or\;\Psi \left(x\right)<e^{-\lambda \varepsilon }\right\}.$$

Based on the remark following (11), it is enough to prove that $X_{H}^{\prime }\subseteq X_{H}$. If $x\in X_{H}^{{}^{\prime }}$, for any given λ∈(0,1), there are only two possible cases: either $\Lambda \left(x\right)>e^{(1-\lambda )\varepsilon }$ or $\Lambda \left(x\right)\leq e^{(1-\lambda )\varepsilon }$. If $\Lambda \left(x\right)>e^{(1-\lambda )\varepsilon }$, then $x\in X_{H}$, and our claim is true. If $\Lambda \left(x\right)\leq e^{(1-\lambda )\varepsilon }$, we have $x\in X_{H}^{\prime }\Rightarrow \frac{\Lambda (x)}{\Psi (x)}>e^{\varepsilon }\Rightarrow \Psi \left(x\right)<\Lambda \left(x\right)e^{-\varepsilon }$. We assumed that $\Lambda \left(x\right)\leq e^{(1-\lambda )\varepsilon }$; therefore, $\Psi \left(x\right)<\Lambda \left(x\right)e^{-\varepsilon }\leq e^{(1-\lambda )\varepsilon }e^{-\varepsilon }=e^{-\lambda \varepsilon }$. Since $\Psi \left(x\right)<e^{-\lambda \varepsilon }$, we have $x\in X_{H}$.  □

This proposition shows the result of applying LDP and ALIP in the watchdog mechanism in terms of the privacy–utility trade-off. While both ε-LDP and (λε,(1−λ)ε)-ALIP imply the same LDP privacy budget, LDP results in fewer high-risk symbols compared to ALIP. This needs to be considered when one applies the watchdog mechanism to achieve LDP or ALIP privacy. Although having fewer high-risk symbols provides better utility, it may compromise privacy. In other words, when $X_{H}^{\prime }\subseteq X_{H}$, then the privacy leakage of the partition $\left\{X_{L}^{\prime },X_{H}^{\prime }\right\}$ is greater than or equal to the privacy leakage of the partition $\left\{X_{L},X_{H}\right\}$. As a result, it is possible that ε-LDP cannot achieve the desired (λε,(1−λ)ε)-ALIP privacy level for a given λ.

Watchdog mechanism with *X*-invariant randomisation is a powerful method with low complexity that can be easily applied to instance-wise measures. However, it significantly degrades the utility [25] because *X*-invariant randomisation obfuscates all high-risk symbols together to minimise privacy leakage, with the cost of deteriorating data resolution. In Section 4, we propose subset merging randomisation to enhance the utility of the watchdog mechanism.

#### 3.1.2. Asymmetric Optimal Random Response (AORR)

ORR was proposed in [28] as a localised instance-wise replacement of the privacy funnel [12]. It is the solution to the optimal utility problem subject to ε-LIP or ε-LDP constraints. For ALIP, we propose asymmetric optimal random response (AORR), which is defined as

(12) $$\begin{array}{cc} \; & \max_{\begin{array}{c} P_{X|Y},P_{Y} \end{array}}I\left(X;Y\right)\\ s.t.\; & S-X-Y\\ & e^{-\varepsilon_{l}}\leq \Psi \left(y\right)\;\mathrm{and}\;\Lambda \left(y\right)\leq e^{\varepsilon_{u}},\;\forall y\in Y. \end{array}$$

Privacy constraints in this optimisation problem form a closed, bounded, convex polytope [28]. It has been proved that vertices of this polytope are the feasible candidates that maximise MI and satisfy privacy constraints [7,28,37]. However, the number of vertices grows exponentially in the dimension of the polyhedron, which is |X|(|X|−1) for LDP and $\left(|X|-1\right)$ for LIP. This makes ORR computationally cumbersome for large |X|. Accordingly, [28] suggests some approaches with lower complexity than ORR to avoid vertex enumeration for the larger sizes of X, but this comes at the cost of lower utility.

#### 3.1.3. Numerical Results

Here, we demonstrate the privacy leakage and utility of AORR and the watchdog mechanism under ALIP. For the utility, we use normalised MI (NMI)

$$\mathrm{NMI}=\frac{I(X;Y)}{H(X)}\in \left[0,1\right].$$

It is clear that the maximum possible utility is obtained when *X* is published without randomisation, where Y=X and I(X;Y)=H(X). Thus, I(X;Y)≤H(X) and NMI ≤1.

**Figure 2:**
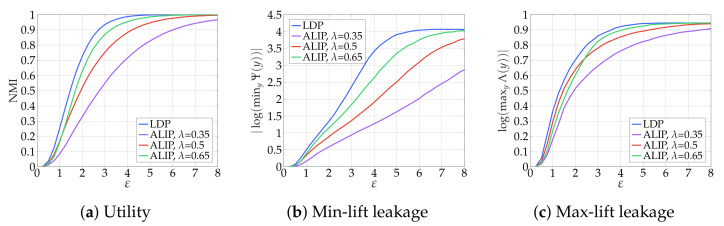
Privacy–utility trade-off of the watchdog mechanism with complete merging randomisation for synthetic data under ε-LDP and $\left(\varepsilon_{l},\varepsilon_{u}\right)$-ALIP, where |X|=17, |S|=5, $\varepsilon_{\mathrm{LDP}}\in \left\{0.25,0.5,0.75,\cdots ,8\right\}$, λ∈{0.35,0.5,0.65}, $\varepsilon_{l}=\lambda \varepsilon$, and $\varepsilon_{u}=\left(1-\lambda \right)\varepsilon$.

**Figure 3:**
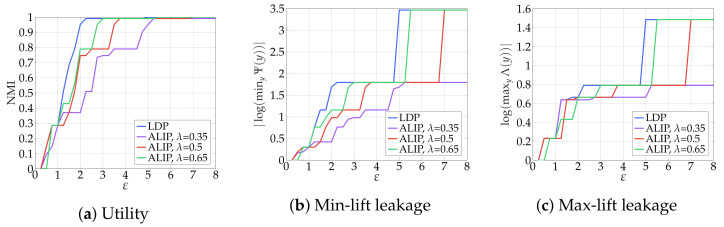
Privacy–utility trade-off of the watchdog mechanism with complete merging randomisation for Adult dataset under ε-LDP and $\left(\varepsilon_{l},\varepsilon_{u}\right)$-ALIP, where S={relationship}, X={occupation}, |X|=15, |S|=5, $\varepsilon_{\mathrm{LDP}}\in \left\{0.25,0.5,0.75,\cdots ,8\right\}$, λ∈{0.35,0.5,0.65}, $\varepsilon_{l}=\lambda \varepsilon$, and $\varepsilon_{u}=\left(1-\lambda \right)\varepsilon$.

**Figure 4:**
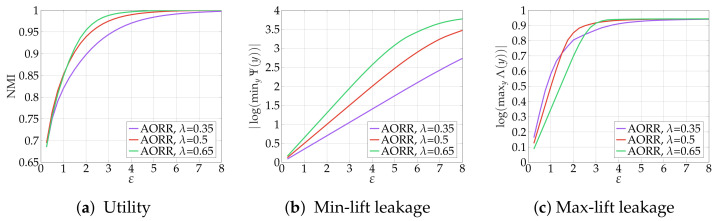
Privacy–utility trade-off of AORR for synthetic data where |X|=17, |S|=5, $\varepsilon_{\mathrm{LDP}}\in \left\{0.25,0.5,0.75,\cdots ,8\right\}$, λ∈{0.35,0.5,0.65}, $\varepsilon_{l}=\lambda \varepsilon$, and $\varepsilon_{u}=\left(1-\lambda \right)\varepsilon$.

**Figure 5:**
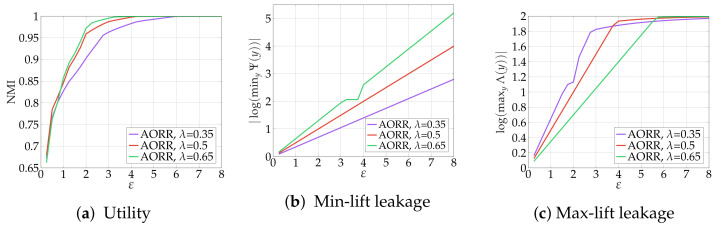
Privacy–utility trade-off of AORR for Adult dataset where S={relationship}, X={occupation}, |X|=15, |S|=5, $\varepsilon_{\mathrm{LDP}}\in \left\{0.25,0.5,0.75,\cdots ,8\right\}$, λ∈{0.35,0.5,0.65}, $\varepsilon_{l}=\lambda \varepsilon$, and $\varepsilon_{u}=\left(1-\lambda \right)\varepsilon$.

We present numerical results for both synthetic and real datasets using MATLAB. For synthetic data, we randomly generated $10^{3}$ distributions for the watchdog mechanism and 100 distributions for the AORR where |X|=17 and |S|=5. These distributions were generated by normalising the output of the *rand* function in MATLAB. For real datasets, we used the Adult dataset [38] and set S={relationship} and X={Occupation}, where |S|=5 and |X|=15. In all scenarios, ε varies from 0.25 to 8, and we consider three cases for $\left(\varepsilon_{u},\varepsilon_{l}\right)$-ALIP, where λ∈{0.35,0.5,0.65}, $\varepsilon_{l}=\lambda \varepsilon$, and $\varepsilon_{u}=\left(1-\lambda \right)\varepsilon$. The results of the watchdog mechanism are shown in Figure 2 and Figure 3 for synthetic and real data, respectively; while the AORR results are presented in Figure 4 and Figure 5. The figures display NMI, $\log\left(\max_{y}\Lambda \left(y\right)\right)$ (max-lift leakage), and $\left|\log\left(\min_{y}\Psi \left(y\right)\right)\right|$ (min-lift leakage) versus the LDP privacy budget ε for real data, and the mean values of the same quantities are shown for synthetic data.

In Figure 2a and Figure 3a, we observe that in the watchdog mechanism, LDP provides higher utility and leakage than ALIP for all values of ε and λ, which confirms Proposition 2. Figure 2a, Figure 3a, Figure 4a and Figure 5a demonstrate that the min-lift relaxation, λ=0.65, enhances utility in the watchdog and AORR mechanisms for ε>1. Note that in all figures, λ=0.5 refers to $\frac{\varepsilon }{2}$-LIP. On the other hand, λ=0.35 results in lower utility. Generally, any value of λ<0.5 reduces utility since it strictly bounds the min-lift while relaxing the max-lift. As the min-lift has a wider range of values, achieving this strict bound enlarges the set $X_{H}$ and requires randomising more symbols, which reduces utility. Another observation here is that AORR incurs significantly higher utility than the watchdog mechanism. For instance, when λ=0.5 and ε=2, the watchdog mechanism results in a utility of 0.52 for synthetic data and 0.73 for the real data, while AORR has a utility of 0.94 and 0.96 for the synthetic and real data, respectively. AORR finds the optimal utility, which, due to PUT, necessarily results in the highest leakage subject to privacy constraints. However, the watchdog mechanism is a nonoptimal solution that minimises leakage of high-risk symbols to provide strong privacy protection, which deteriorates utility. To solve this drawback of the watchdog mechanism, we propose a subset randomisation method in the following section.

## 4. Subset Merging in Watchdog Mechanism

The watchdog mechanism with *X*-invariant randomisation is a low-complexity method that can be easily applied when the privacy measures are symbol-wise. *X*-invariant randomisation is the optimal privacy protection for the high-risk symbols that minimises privacy leakage in $X_{H}$ and necessarily results in the worst data resolution. Thus, in this section, we propose the *subset merging* algorithm to improve data resolution by randomising disjoint subsets of high-risk symbols and enhancing utility in the watchdog mechanism. In the following, we show that applying *X*-invariant randomisation to disjoint subsets of $X_{H}$ increases the utility.

Let $G_{X_{H}}=\left\{X_{1},X_{2},\cdots X_{g}\right\}$ be a partition of $X_{H}$, where for every i∈[g], $X_{i}\subseteq X_{H}$: $X_{i}\cap X_{j}=⌀,i\neq j$, and $X_{H}=\cup_{i=1}^{g}X_{i}$. We randomise each subset $X_{i}\in G_{X_{H}}$ by *X*-invariant randomisation $R_{Y_{i}}\left(y\right)$ for $x\in X_{i}$ and $y\in Y_{i}$, where $\sum_{y\in Y_{i}}R_{Y_{i}}\left(y\right)=1$. The resulting MI between *X* and *Y* is


(13) $$I\left(X;Y\right)=H\left(X\right)-\sum_{i=1}^{g}\sum_{x\in X_{i}}P_{X}\left(x\right)\log\frac{P(X_{i})}{P_{X}\left(x\right)}.$$

**Definition** **4.** *Assume two partitions, $G_{X_{H}}=\left\{X_{1},\cdots ,X_{g}\right\}$ and $G_{X_{H}}^{{}^{\prime }}=\left\{X_{1}^{{}^{\prime }},\cdots ,X_{g^{\prime }}^{{}^{\prime }}\right\}$. We say that $G_{X_{H}}^{{}^{\prime }}$ is a refinement of $G_{X_{H}}$, or $G_{X_{H}}$ is an aggregation of $G_{X_{H}}^{{}^{\prime }}$, if for every i∈[g], $X_{i}=\cup_{j\in J_{i}}X_{j}^{{}^{\prime }}$ where $J_{i}\subseteq \left[g^{\prime }\right]$, and $P\left(X_{i}\right)=\sum_{j\in J_{i}}P(X_{j}^{{}^{\prime }})$ (this definition is inspired from [39] (Definition 10)).*

If $G_{X_{H}}^{{}^{\prime }}$ is a refinement of $G_{X_{H}}$, then $I_{G_{X_{H}}}\left(X;Y\right)\leq I_{G_{X_{H}}^{{}^{\prime }}}\left(X;Y\right)$.

Obtaining the optimal $G_{X_{H}}$ that maximises utility and satisfies privacy constraints is a combinatorial optimisation problem over all possible partitions of $X_{H}$, which is cumbersome to solve. Therefore, we propose a heuristic method in the following.

### 4.1. Greedy Algorithm to Make Refined Subsets of High-Risk Symbols

In Algorithm 1, we propose a bottom-up algorithm that constitutes a partition of $X_{H}$ by merging high-risk symbols in disjoint subsets. It works based on a leakage risk metric for each $x\in X_{H}$: ω(x)=Λ(x)+Ψ(x) for ALIP and ω(x)=Γ(x) for LDP. For LIP, ω(x)=max{logΛ(x),|logΨ(x)|}. This metric is used to order the subsets by the privacy risk level. Accordingly, to constitute a subset $X_{i}\subseteq X_{H}$, Algorithm 1 bootstraps from the highest risk symbol $X_{i}=\left\{\arg\;\max_{x\in X_{H}}\omega \left(x\right)\right\}$ (line 5). Then, it merges a symbol $x^{*}$ with $X_{i}$ that minimises $\omega (X_{i}\cup x^{*})$ (line 7), as long as the privacy constraints are satisfied in $X_{i}$ (line 6). The ALIP privacy constraints for a subset $X_{i}$ are given by

(14) $$e^{-\varepsilon_{l}}\leq \Psi \left(X_{i}\right)\;and\;\Lambda \left(X_{i}\right)\leq e^{\varepsilon_{u}},$$

where $\Psi \left(X_{i}\right)=\min_{s\in S}\frac{\sum_{x\in X_{i}}P_{X|S}\left(x|s\right)}{\sum_{x\in X_{i}}P_{X}\left(x\right)}$ and $\Lambda \left(X_{i}\right)=\max_{s\in S}\frac{\sum_{x\in X_{i}}P_{X|S}\left(x|s\right)}{\sum_{x\in X_{i}}P_{X}\left(x\right)}$. For LDP constraint, we have $\Gamma \left(X_{i}\right)=\frac{\Lambda (X_{i})}{\Psi (X_{i})}\leq e^{\varepsilon }$. In Algorithm 1, we used ALIP privacy constraints for the while loops condition in lines 4, 6, and 12. For LDP, the privacy constraint is changed to $\Gamma (X_{Q})>\varepsilon$, and ω(x) for LDP is applied. After the constitution of the partition $G_{X_{H}}$, the last subset $X_{g}$ may not meet privacy constraints. Therefore, the leakage of $X_{g}$ is checked (line 12), and if there is a privacy breach, an agglomerate $X_{g}$ is made by merging other subsets to it that minimises subset risk, $\omega \left(X_{g}\right)=\Lambda \left(X_{g}\right)+\Psi \left(X_{g}\right)$ (lines 13–14), until privacy constraints are satisfied.

**Algorithm 1:** Subset merging in the watchdog mechanism.

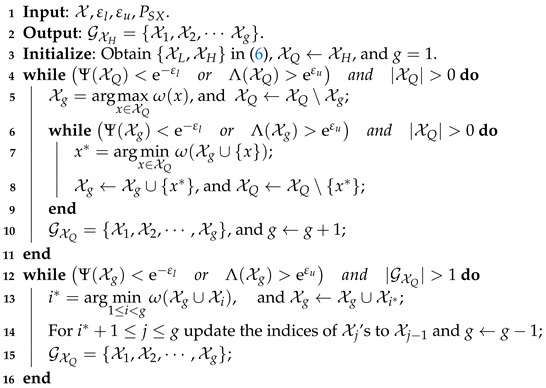



### 4.2. Numerical Results

**Figure 6:**
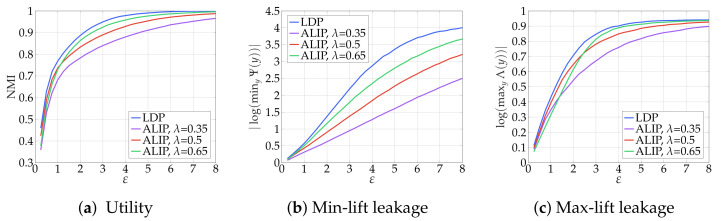
Privacy–utility trade-off of subset merging randomisation under ε-LDP and $\left(\varepsilon_{l},\varepsilon_{u}\right)$-ALIP, where |X|=17, |S|=5, $\varepsilon_{\mathrm{LDP}}\in \left\{0.25,0.5,0.75,\cdots ,8\right\}$, λ∈{0.35,0.5,0.65}, $\varepsilon_{l}=\lambda \varepsilon$, and $\varepsilon_{u}=\left(1-\lambda \right)\varepsilon$.

**Figure 7:**
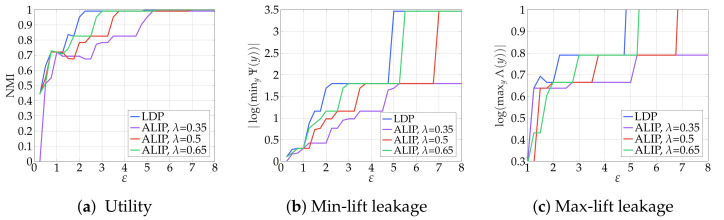
Privacy–utility trade-off of subset merging randomisation for Adult dataset under ε-LDP and $\left(\varepsilon_{l},\varepsilon_{u}\right)$-ALIP, where S={relationship}, X={occupation}, |X|=15, |S|=5, $\varepsilon_{\mathrm{LDP}}\in \left\{0.25,0.5,0.75,\cdots ,8\right\}$, λ∈{0.35,0.5,0.65}, $\varepsilon_{l}=\lambda \varepsilon$, and $\varepsilon_{u}=\left(1-\lambda \right)\varepsilon$.

We show PUT for ALIP and LDP under subset merging randomisation in Figure 6 and Figure 7 for synthetic and real data, respectively, with the same setup for the watchdog mechanism in Section 3.1.3. Compared with the complete merging (Figure 2 and Figure 3), the utility was enhanced significantly for both LDP and ALIP in all scenarios under the same privacy constraint. For instance, consider the symmetric case λ=0.5 when ε=1, and compare PUT between the subset and complete merging. Figure 6a and Figure 7a demonstrate a utility value of around 0.73 for the subset merging compared to the utilities of 0.17 and 0.28 for the complete merging in Figure 2a and Figure 3a, which are almost 320% and 160% utility enhancement. Moreover, as Figure 6b,c and Figure 7b,c illustrate, privacy constraints are satisfied in all cases.

## 5. Subset Random Response

In the previous section, we showed that subset merging enhances utility in the watchdog mechanism significantly. In this section, we propose a method to decrease the complexity of AORR for large datasets. We adopt AORR for subsets of $X_{H}$ to decrease the complexity of AORR for large sets such that random response becomes applicable for typically an order of magnitude larger X.

The AORR optimisation problem in (12) is equivalent to the following problem:

(15) $$\begin{array}{cc} \; & H\left(x\right)-\min_{P_{X|Y},P_{Y}}H\left(X|Y\right)\\ & s.t.\;S-X-Y\\ & \;e^{-\varepsilon_{l}}\leq \Psi \left(y\right)\;\mathrm{and}\;\Lambda \left(y\right)\leq e^{\varepsilon_{u}},\;\forall y\in Y. \end{array}$$

To reduce the complexity of (15), we divide X into $X_{L}$ and $X_{H}$, similar to the watchdog mechanism, and make a partition $G_{X_{H}}=\left\{X_{1},X_{2},\cdots X_{g}\right\}$ from $X_{H}$. We randomise each subset $X_{i}\in G_{X_{H}}$, i∈[g], separately by a randomisation pair $\left(Q_{i},q_{i}\right)$, where $Q_{i}$ is a matrix in $R^{|X_{i}\left|\times \right|Y_{i}|}$ and $q_{i}$ is a vector in $R^{|Y_{i}|}$.

The elements of $Q_{i}$ and $q_{i}$ are given by

(16) $$\begin{array}{c} Q_{i}\left(x|y\right)=\mathrm{Pr}\left[X=x|Y=y\right],\;x\in X_{i},y\in Y_{i}, \end{array}$$

(17) $$\begin{array}{c} q_{i}\left(y\right)=\mathrm{Pr}\left[Y=y\right],\;y\in Y_{i}. \end{array}$$

For each $y\in Y_{i}$, we have $\sum_{x\in X_{i}}Q_{i}\left(x|y\right)=1$. Consequently, $H\left(X|Y\right)=\sum_{i\in [g]}H_{i}\left(X|Y\right)$, where

(18) $$H_{i}\left(X|Y\right)=-\sum_{y\in Y_{i}}q_{i}\left(y\right)\sum_{x\in X_{i}}Q_{i}\left(x|y\right)\log Q_{i}\left(x|y\right),\;i\in \left[g\right].$$

This setting turns (15) into *g* optimisation problems for each subset $X_{i}\in G_{X_{H}},$i∈[g] as follows:

(19) $$\begin{array}{cc} \; & \min_{Q_{i},q_{i}}H_{i}\left(X|Y\right) \end{array}$$

(20) $$\begin{array}{cccc} s.t.\; & 0\leq q_{i}\left(y\right), & \; & \forall y\in Y_{i}, \end{array}$$

(21) $$\begin{array}{cccc} \; & 0\leq Q_{i}\left(x|y\right), & \; & \forall x\in X_{i},\forall y\in Y_{i}, \end{array}$$

(22) $$\begin{array}{cccc} \; & \sum_{x\in X_{i}}Q_{i}\left(x|y\right)=1, & \; & \forall y\in Y_{i}, \end{array}$$

(23) $$\begin{array}{cccc} \; & \sum_{y\in Y_{i}}Q_{i}\left(x|y\right)q_{i}\left(y\right)=P_{X}\left(x\right), & \; & x\in X_{i}, \end{array}$$

(24) $$\begin{array}{cccc} \; & e^{-\varepsilon_{l}}P_{S}\left(s\right)\leq \sum_{x\in X_{i}}P_{S|X}\left(s|x\right)Q_{i}\left(x|y\right)\leq e^{\varepsilon_{u}}P_{S}\left(s\right), & \; & \forall s\in S,y\in Y_{i}. \end{array}$$

The columns of the randomisation matrix $Q_{i}$, i∈[g] can be expressed as the members of a convex and bounded polytope $\Pi_{i}$, which is given by the following constraints:

(25) $$\Pi_{i}=\left\{\begin{array}{cc} \; & v\in R^{|X_{i}|}:\\ \; & 0\leq v_{k},\forall k\in \left[\right|X_{i}\left|\right],\\ \; & \sum_{k=1}^{|X_{i}|}v_{k}=1,\\ \; & e^{-\varepsilon_{l}}P_{S}\left(s\right)\leq \sum_{x\in X_{i}}P_{S|X}\left(s|x\right)v_{k}\leq e^{\varepsilon_{u}}P_{S}\left(s\right),\forall s\in S,k\in \left[\right|X_{i}\left|\right] \end{array}\right\}.$$

For each $X_{i}\in G_{X_{H}},$ i∈[g], let $V_{i}=\left\{v_{1}^{i}\cdots ,v_{M}^{i}\right\}$ be the vertices of $\Pi_{i}$ in (25), $H\left(v_{k}^{i}\right)$ be the entropy of each $v_{k}^{i}$ for k∈[M], $P_{X}^{i}$ be the probability vector of $x\in X_{i}$, and $\beta^{i}$ be the solution to the following optimisation:

(26) $$\begin{array}{cc} \; & \min_{\beta^{i}\in R^{M}}\sum_{k=1}^{M}H\left(v_{k}^{i}\right)\beta_{k}^{i},\\ s.t.\; & \beta_{k}^{i}\geq 0,\;\forall k\in \left[M\right],\\ \; & \sum_{k=1}^{M}v_{k}^{i}\beta_{k}^{i}=P_{X}^{i}. \end{array}$$

Then, $Y_{i}$ and $\left(Q_{i},q_{i}\right)$ are given by

(27) $$\begin{array}{c} Y_{i}=\left\{y:\beta_{y}^{i}\neq 0\right\}; \end{array}$$

(28) $$\begin{array}{c} q_{i}\left(y\right)=\beta_{y}^{i}\;\mathrm{and}\;Q_{i}\left(.|y\right)=v_{y}^{i},\;y\in Y_{i}. \end{array}$$

The $\left(\varepsilon_{l},\varepsilon_{u}\right)$-ALIP protocol, M:X→Y, is given by the pair of $\left(P_{X|Y},P_{Y}\right)$ as follows:

(29) $$P_{X|Y}=\left\{\begin{array}{cc} 1_{\left\{x=y\right\}}, & x,y\in X_{L}=Y_{L},\\ Q_{i}\left(x|y\right), & x\in X_{i},y\in Y_{i},i\in \left[g\right],\\ 0, & otherwise; \end{array}\right.$$

(30) $$P_{Y}=\left\{\begin{array}{cc} P_{X}\left(y\right), & y\in Y_{L},\\ q_{i}\left(y\right), & y\in Y_{i},i\in \left[g\right]. \end{array}\right.$$

### 5.1. Algorithm for Subset Random Response

We propose Algorithm 2 to implement AORR for subsets of $X_{H}$, which we call *subset random response* (SRR). In this algorithm, first, we obtain a partition $G_{X_{H}}=\left\{X_{1},X_{2},\cdots \;X_{g}\right\}$ of $X_{H}$ via Algorithm 1. Then, based on $G_{X_{H}}$, we make another partition $O_{X_{H}}$ and find the optimal random response for each subset $X_{i}^{{}^{\prime }}\in O_{X_{H}}$ (line 5). By obtaining the optimal random responses for all subsets, we obtain a pair $\left(Q_{i},q_{i}\right)$ for each subset $X_{i}^{{}^{\prime }}$, and consequently $P_{X|Y}$ and $P_{Y}$, by (29) and (30) (lines 20–23). The while loop in lines 7–14 is for the particular cases when the polytope in (25) is empty for a subset $X_{i}^{\prime }$. It may occur for strict privacy conditions where the privacy budget is very small. Since we reduce the dimension of the original polytope in (15), it increases the possibility that no feasible random response exists in some cases. Therefore, in such cases, we make a union with other subsets until we have a nonempty polytope. If $|G_{X_{H}}|>0$, then we make a union with another subset in $G_{X_{H}}$ (line 9). If $|G_{X_{H}}|=0$, it means that $X_{g^{\prime }}^{\prime }$ is the last subset in $O_{X_{H}}$; therefore, we make a union with previously made subsets in $O_{X_{H}}$ and update the index $g^{\prime }$ (line 12). Then, if the condition in lines 17–18 is for the cases where there is no feasible polytope after making a union of all subsets, this means that the problem in (15) cannot be solved. Whenever this occurs, we apply the subset merging mechanism.

The number of polytope vertices in AORR is $n∼O\left(\exp\left(|X|-1\right)\right)$, and the time complexity is O(n). As |X| increases, the complexity of AORR increases exponentially in the |X|−1. In SRR, for each $X_{i}\in G_{X_{H}}$, i∈[g], the number of vertices is $n_{i}∼O\left(\exp\left(|X_{i}|-1\right)\right)$, and the complexity of SRR is $O(\max_{i}n_{i})$.

**Algorithm 2:** Subset random response.

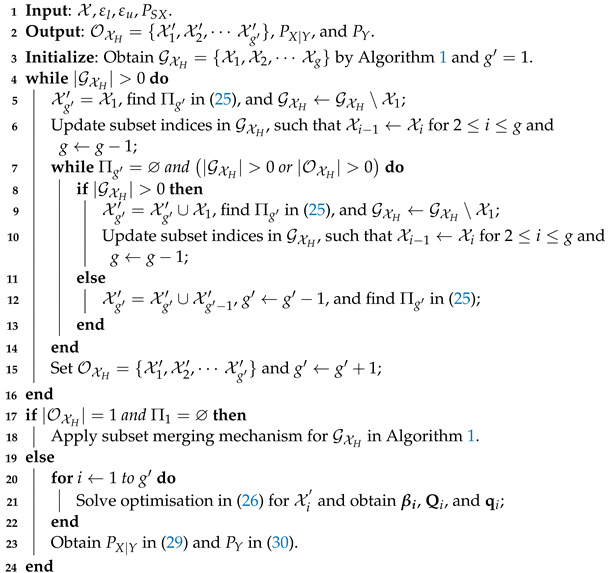



### 5.2. Numerical Results

**Figure 8:**
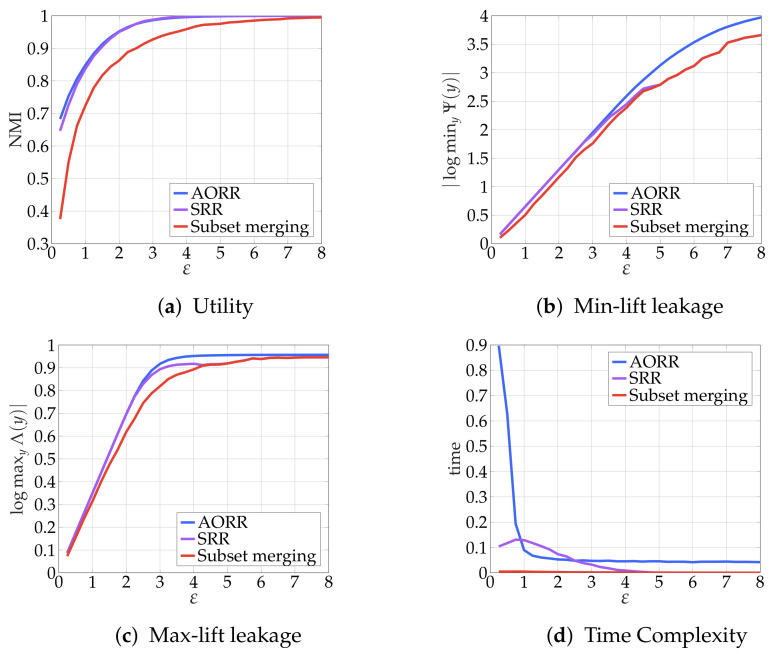
Comparison of privacy–utility trade-off and time complexity between AORR, SRR (Algorithm 2), and subset merging (Algorithm 1) for synthetic data, where |X|=17, |S|=5, $\varepsilon_{\mathrm{LDP}}\in \left\{0.25,0.5,0.75,\cdots ,8\right\}$, λ=0.65, $\varepsilon_{l}=\lambda \varepsilon_{\mathrm{LDP}}$, and $\varepsilon_{u}=\left(1-\lambda \right)\varepsilon_{\mathrm{LDP}}$.

**Figure 9:**
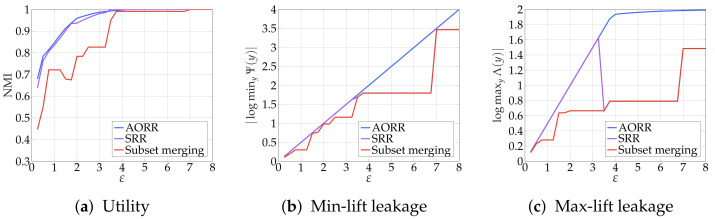
Comparison of privacy–utility trade-off between AORR, SRR (Algorithm 2), and subset merging (Algorithm 1) for Adult dataset, where S={relationship}, X={occupation}, |X|=15, |S|=5, $\varepsilon_{\mathrm{LDP}}\in \left\{0.25,0.5,0.75,\cdots ,8\right\}$, λ=0.65, $\varepsilon_{l}=\lambda \varepsilon_{\mathrm{LDP}}$, and $\varepsilon_{u}=\left(1-\lambda \right)\varepsilon_{\mathrm{LDP}}$.

Here, we compare the PUT of AORR with SRR in Algorithm 2 and subset merging in Algorithm 1. Figure 8 depicts mean values of utility, leakage, and time complexity for 100 randomly generated distributions where λ=0.65 and simulation setup is the same as that in Section 3.1.3. The result of the Adult dataset is also shown in Figure 9.

**Figure 10:**
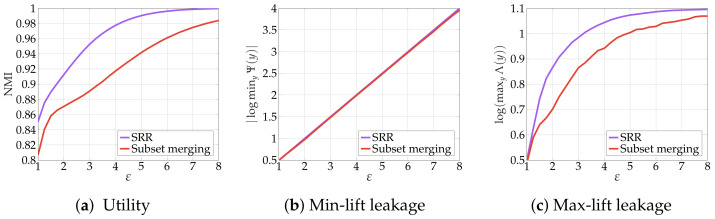
Comparison of privacy–utility trade-off and time complexity between SRR (Algorithm 2) and subset merging (Algorithm 1) for synthetic data, where |X|=200, |S|=15, $\varepsilon_{\mathrm{LDP}}\in \left\{1,1.25,1.5,1.75,\cdots ,8\right\}$, and $\varepsilon_{l}=\varepsilon_{u}=\frac{\varepsilon_{\mathrm{LDP}}}{2}$.

Figure 8a–c and Figure 9 demonstrate that SRR results in better utility and higher leakage than subset merging, and its PUT is very close to AORR. Figure 8d illustrates the processing time of each mechanism for synthetic data from which we observe that the complexity of AORR and SRR is much higher than the subset merging. Running SRR is less complex than AORR for strict privacy constraints (ε<1) and for ε>2.5. While SRR shows higher complexity for some privacy budgets, 1≤ε≤2.5, it has the advantage in high-dimension systems. Figure 10 shows a PUT comparison between SRR and subset merging for synthetic data where |X|=200, |S|=15, ε∈{1,1.25,⋯,8}, and λ=0.5. This experiment shows that both SRR and subset merging are applicable to large datasets. Obviously, SRR provides better utility (Figure 10a) and higher leakage (Figure 10b,c), which is still below the given budgets $\varepsilon_{l}$ and $\varepsilon_{u}$.

## 6. Lift-Based and Lift-Inverse Measures

In this section, we consider some recently proposed privacy measures that quantify the divergence between the posterior and prior belief on sensitive features, including $\ell_{1}$-norm [32], strong $\chi^{2}$-privacy criterion [33], and α-lift [34]. They have been proposed as a stronger version of their corresponding average measures, which are the total variation distance [35], $\chi^{2}$-divergence [40], and Sibson MI [16], respectively. We call them *lift-based* measures and define them in the following.

**Definition** **5.** *For each y∈Y, lift-based privacy measures are defined as follows:*

- *The $\ell_{1}$-lift is given by*
(31) $$\Lambda_{\ell_{1}}\left(y\right)≜\sum_{s\in S}P_{S}\left(s\right)\left|l\left(s,y\right)-1\right|,$$
  *then the total variation distance will be*
$$T\left(S;Y\right)=\frac{1}{2}E_{Y}\left[\Lambda_{\ell_{1}}\left(Y\right)\right].$$
- *The $\chi^{2}$-lift is given by*
(32) $$\Lambda_{\chi^{2}}\left(y\right)≜\sum_{s\in S}P_{S}\left(s\right){\left(l(s,y)-1\right)}^{2},$$
  *then $\chi^{2}$-divergence will be*
$$\chi^{2}\left(S;Y\right)=E_{Y}\left[\Lambda_{\chi^{2}}\left(Y\right)\right].$$
- *The α-lift is given by*
(33) $$\Lambda_{\alpha }^{S}\left(y\right)≜{\left(\sum_{s\in S}P_{S}\left(s\right)l{\left(s,y\right)}^{\alpha }\right)}^{1/\alpha },$$
  *then Sibson MI will be*
$$I_{\alpha }^{S}\left(S;Y\right)=\frac{\alpha }{\alpha -1}\log E_{Y}\left[\Lambda_{\alpha }^{S}\left(Y\right)\right].$$

Here, we reveal their relationship with ALIP.

**Proposition** **3.** *If $\left(\varepsilon_{l},\varepsilon_{u}\right)$-ALIP is satisfied, then*

 *1.*  *$\max_{y\in Y}\Lambda_{\ell_{1}}\left(y\right)\leq e^{\varepsilon_{u}}-1$;*
 *2.*  *$\max_{y\in Y}\Lambda_{\chi^{2}}\left(y\right)\leq {\left(e^{\varepsilon_{u}}-1\right)}^{2}$;*
 *3.*  *$\max_{y\in Y}\Lambda_{\alpha }^{S}\left(y\right)\leq e^{\varepsilon_{u}}$.*

The proof is given in Appendix D.

**Proposition** **4.** *Let $s_{y}=\arg\;\max_{s\in S}\left[l\left(s,y\right)\right]$ and $\bar{y}=\arg\;\max_{y\in Y}\Lambda \left(y\right)$, then*

 *1.*  *If $\max_{y\in Y}\Lambda_{\ell_{1}}\left(y\right)\leq \varepsilon \Rightarrow \max_{y\in Y}\Lambda \left(y\right)\leq \varepsilon /P_{S}\left(s_{\bar{y}}\right)+1$;*
 *2.*  *If $\max_{y\in Y}\Lambda_{\chi^{2}}\left(y\right)\leq \varepsilon \Rightarrow \max_{y\in Y}\Lambda \left(y\right)\leq \sqrt{\varepsilon /P_{S}\left(s_{\bar{y}}\right)}+1$;*
 *3.*  *If $\max_{y\in Y}\Lambda_{\alpha }^{S}\left(y\right)\leq \varepsilon \Rightarrow \max_{y\in Y}\Lambda \left(y\right)\leq \varepsilon /P_{S}{\left(s_{\bar{y}}\right)}^{\frac{1}{\alpha }}$.*

**Proof.**  The proof is given in Appendix E. □

Proposition 3 shows that lift-based measures, similar to their corresponding average leakages, are upper-bounded by the max-lift bound. Proposition 4 indicates that if we bound lift-based measures, they can only restrict the max-lift leakage. Accordingly, if one only applies a lift-based measure to protect privacy, such as in previous works [32,33,34], it may cause significant leakage on the min-lift. Therefore, in the following, we propose lift-inverse measures to bound the min-lift leakage.

### 6.1. Lift-Inverse Measures

In Propositions 3 and 4, we showed that lift-based measures only bound the max-lift. In this subsection, we present lift-inverse measures to restrict the min-lift leakage, $\min_{y\in Y}\Psi \left(y\right)=\min_{y\in Y}[\min_{s\in S}l\left(s,y\right)$].

**Definition** **6.** *For lift-based measures in *(31)* to *(33)*, we replace l(s,y) with $\frac{1}{l(s,y)}$ and call the resulting quantities lift-inverse measures.*

- *The $\ell_{1}$-lift-inverse is given by*
(34) $$\Psi_{\ell_{1}}\left(y\right)=\sum_{s\in S}P_{S}\left(s\right)\left|\frac{1}{\ell (s,y)}-1\right|.$$
- *The $\chi^{2}$-lift-inverse is given by*
(35) $$\Psi_{\chi^{2}}\left(y\right)≜\sum_{s\in S}P_{S}\left(s\right){\left(\frac{1}{\ell (s,y)}-1\right)}^{2}.$$
- *The α-lift-inverse is given by*
(36) $$\Psi_{\alpha }^{S}\left(y\right)≜{\left(\sum_{s\in S}P_{S}\left(s\right){\left(\frac{1}{\ell (s,y)}\right)}^{\alpha }\right)}^{1/\alpha }.$$

In the following propositions, we show the relationship between $\left(\varepsilon_{l},\varepsilon_{u}\right)$-ALIP.

**Proposition** **5.** *If $\left(\varepsilon_{l},\varepsilon_{u}\right)$-ALIP is achieved, we have*

 *1.*  *
$\max_{y\in Y}\Psi_{\ell_{1}}\left(y\right)\leq e^{\varepsilon_{l}}-1;$
*
 *2.*  *
$\max_{y\in Y}\Psi_{\chi^{2}}\left(y\right)\leq {\left(e^{\varepsilon_{l}}-1\right)}^{2};$
*
 *3.* 
$\max_{y\in Y}\Psi_{\alpha }^{S}\left(y\right)\leq e^{\varepsilon_{l}}.$

**Proof.**  The proof is provided in Appendix F. □

**Proposition** **6.** *Let $s_{y}=\arg\;\min_{s}l\left(s,y\right)$ and $\underline{y}=\arg\;\min_{y}\left[\Psi \left(y\right)\right]$, then*

 *1.*  *If $\max_{y\in Y}\Psi_{\ell_{1}}\left(y\right)\leq \varepsilon \Rightarrow \min_{y\in Y}\Psi \left(y\right)\geq \frac{P_{S}\left(s_{\underline{y}}\right)}{\varepsilon +P_{S}\left(s_{\underline{y}}\right)};$*
 *2.*  *If $\max_{y\in Y}\Psi_{\chi^{2}}\left(y\right)\leq \varepsilon \Rightarrow \min_{y\in Y}\Psi \left(y\right)\geq \frac{\sqrt{P_{S}\left(s_{\underline{y}}\right)}}{\sqrt{\varepsilon }+\sqrt{P_{S}\left(s_{\underline{y}}\right)}};$*
 *3.*  *If $\max_{y\in Y}\Psi_{\alpha }^{S}\left(y\right)\leq \varepsilon \Rightarrow \min_{y\in Y}\Psi \left(y\right)\geq \varepsilon^{-1}P_{S}{\left(s_{\underline{y}}\right)}^{\frac{1}{\alpha }}.$*

**Proof.**  The proof is provided in Appendix G. □

Propositions 5 and 6 demonstrate that the aforementioned lift-inverse measures are associated with the min-lift, and bounding them can restrict the min-lift leakage. Since lift-based and lift-inverse measures quantify privacy leakage by a function of lift averaged over sensitive features, they can be regarded as more relaxed measures than the min- and max-lifts.

### 6.2. PUT and Numerical Results

Optimal randomisations for $\ell_{1}$-lift and $\chi^{2}$-lift privacy, which maximise MI as the utility measure, were proposed in [32,33], respectively. Note that ORR is not applicable to these measures since their privacy constraints are not convex. However, here, we apply the watchdog mechanism with subset merging randomisation to investigate the PUT for lift-based and lift-inverse measures. This application shows that the watchdog mechanism with *X*-invariant randomisation is a low-complexity method that can be applied to all the aforementioned measures. Moreover, our subset merging algorithm significantly enhances the utility, which is comparable to the optimal solutions.

To apply lift-based and lift-inverse measures to the subset merging algorithm, we replace Λ(y) and Ψ(y) in (6) and Algorithm 1, with the corresponding lift-based and lift-inverse measures in Definitions 5 and 6, respectively. For example, $X_{L}$ and $X_{H}$ for the α-lift are obtained as

(37) $$\begin{array}{c} X_{L}≜\left\{x\in X:\Psi_{\alpha }^{S}\left(x\right)\leq e^{\varepsilon_{l}}\;and\;\Lambda_{\alpha }^{S}\left(x\right)\leq e^{\varepsilon_{u}}\right\}\;and\;X_{H}=X\backslash X_{L}. \end{array}$$

The privacy risk measure in Algorithm 1 is also given by $\omega \left(x\right)=\Psi_{\alpha }^{S}\left(x\right)+\Lambda_{\alpha }^{S}\left(x\right)$. We compare the PUT of lift-based and lift-inverse privacy with ($\varepsilon_{l},\varepsilon_{u}$)-ALIP, where lift-based and lift-inverse measures are bounded as follows:

- $\ell_{1}$-privacy: $\Lambda_{\ell_{1}}\left(y\right)\;\leq e^{\varepsilon_{u}}-1$          and $\;\Psi_{\ell_{1}}\left(y\right)\leq e^{\varepsilon_{l}}-1,\;\forall y\in Y.$
- $\chi^{2}$-privacy: $\Lambda_{\chi^{2}}\left(y\right)\leq {\left(e^{\varepsilon_{u}}-1\right)}^{2}$    and $\;\Psi_{\chi^{2}}\left(y\right)\;\leq {\left(e^{\varepsilon_{l}}-1\right)}^{2},\;\;\;\forall y\in Y.$
- α-lift-privacy: $\Lambda_{\alpha }^{S}\left(y\right)\;\leq e^{\varepsilon_{u}}$              and $\;\Psi_{\alpha }^{S}\left(y\right)\;\leq e^{\varepsilon_{l}},\;\forall y\in Y.$

**Figure 11:**
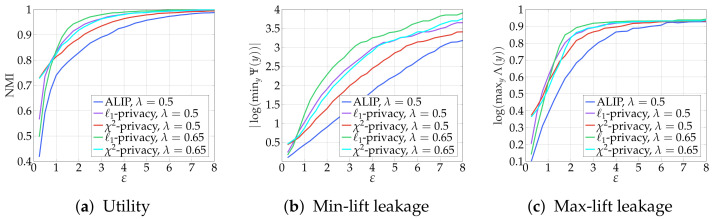
Comparison of privacy–utility trade-off between ALIP, $\ell_{1}$-privacy, and $\chi^{2}$-privacy for synthetic data, where |X|=17, |S|=5, $\varepsilon_{\mathrm{LDP}}\in \left\{0.25,0.5,0.75,\cdots ,8\right\}$, λ∈{0.5,0.65}, $\varepsilon_{l}=\lambda \varepsilon$, and $\varepsilon_{u}=\left(1-\lambda \right)\varepsilon$.

**Figure 12:**
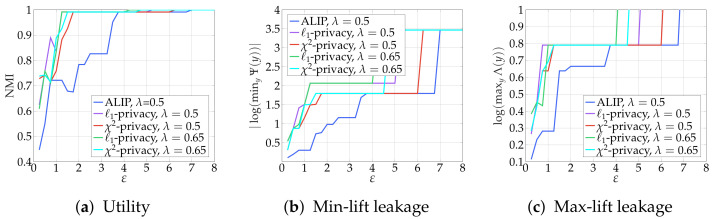
Comparison of privacy–utility trade-off between ALIP, $\ell_{1}$-privacy, and $\chi^{2}$-privacy for Adult dataset where, S={relationship}, X={occupation}, |X|=15, |S|=5, $\varepsilon_{\mathrm{LDP}}\in \left\{0.25,0.5,0.75,\cdots ,8\right\}$, λ∈{0.5,0.65}, $\varepsilon_{l}=\lambda \varepsilon$, and $\varepsilon_{u}=\left(1-\lambda \right)\varepsilon$.

We apply the subset merging mechanism with the simulation setup in Section 3.1.3. Figure 11 and Figure 12 demonstrate the PUT of ALIP for λ=0.5 and $\ell_{1}$ and $\chi^{2}$ privacy for λ={0.5,0.65} for synthetic and Adult dataset, respectively. When λ=0.5, $\ell_{1}$ and $\chi^{2}$ privacy result in higher utility compared to ALIP for all values of ε since lift-based and lift-inverse measures are relaxations of max- and min-lift. To observe the effect of the asymmetric scenario, we depict $\ell_{1}$ and $\chi^{2}$ privacy for λ=0.65. From Figure 11a and Figure 12a, we observe that lift-inverse relaxation (λ=0.65) enhances utility significantly for ε>1, but worsens utility for ε<1. The reason is that when ε<1, lift-inverse privacy constraint in the asymmetric scenario is strict, which requires more symbols to be merged in each subset and causes larger subsets and utility degradation.

**Figure 13:**
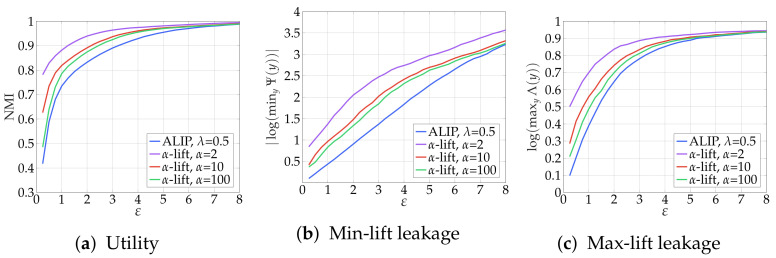
Comparison of privacy–utility trade-off between ALIP and α-lift-privacy, where |X|=17, |S|=5, $\varepsilon_{\mathrm{LDP}}\in \left\{0.25,0.5,0.75,\cdots ,8\right\}$, $\varepsilon_{l}=\varepsilon_{u}=\frac{\varepsilon_{\mathrm{LDP}}}{2}$, and α∈{2,10,100}.

A comparison between α-lift privacy and ALIP for synthetic data is shown in Figure 13 for α∈{2,10,100}. α-lift privacy is tunable such that when α=∞, it is equivalent to ALIP, and when α<∞, it results in a relaxation scenario. We observe tunable property in Figure 13 where α=2 has the highest utility. Moreover, when α increases, the PUT of α-lift privacy becomes closer to ALIP.

## 7. Conclusions

In this paper, we studied lift, the likelihood ratio between posterior and prior belief about sensitive features in a dataset. We demonstrated the distinction between the min- and max-lifts in terms of data privacy concerns. We proposed ALIP as a generalised version of LIP to have a more compatible notion of privacy with lift asymmetry. ALIP can enhance utility in the watchdog and ORR mechanisms, two main approaches to achieve lift-based privacy. We proposed two subset randomisation methods to enhance the utility of the watchdog mechanism and reduce ORR complexity for large datasets. We also investigated the existing lift-based measures, showing that they could incur significant leakage on the min-lift. Thus, we proposed lift-inverse measures to restrict the min-lift leakage. Finally, we applied the watchdog mechanism to study the PUT of lift-based and lift-inverse measures. For future work, one can consider the applicable operational meaning of the min-lift and max-lift. Subset randomisation can be applied to decrease the complexity and enhance the utility of other privacy mechanisms. Moreover, optimal randomisation for α-lift is also unknown and could be considered.

## Data Availability

For real-world data simulations we have used public Adult dataset from UCI machine learning repository https://archive-beta.ics.uci.edu/dataset/2/adult.

## Appendix A

In this appendix, we represent the privacy breach in terms of min-lift and max-lift based on the work of Evfimievsk et al. [29]. In this work, they proposed the following definition for privacy breach and provided a detailed example [29] (Example 1).

**Definition** **A1.**
*For $\rho_{1},\rho_{2}\in R_{+}:0<\rho_{1}<\rho_{2}<1$, we say that there is a straight or upward $\rho_{1}$-to-$\rho_{2}$ privacy breach with respect to a property Q(S) if for some y∈Y*

$$\mathrm{Pr}\left[Q\left(S\right)\right]\leq \rho_{1},\;\mathrm{Pr}\left[Q\left(S\right)|Y=y\right]>\rho_{2}.$$

*We say that there is a downward $\rho_{2}$-to-$\rho_{1}$ privacy breach with respect to Q(S) if for some y∈Y*

$$\mathrm{Pr}\left[Q\left(S\right)\right]\geq \rho_{2},\;\mathrm{Pr}\left[Q\left(S\right)|Y=y\right]<\rho_{1}.$$

*Using the property $Q^{\prime }\left(S\right)=\neg Q\left(S\right)$, we have*

$$\mathrm{Pr}\left[Q^{\prime }\left(S\right)\right]\leq 1-\rho_{2},\;\mathrm{Pr}\left[Q^{\prime }\left(S\right)|Y=y\right]\geq 1-\rho_{1}.$$

Therefore, a downward $\rho_{2}$-to-$\rho_{1}$ privacy breach implies an upward $\left(1-\rho_{2}\right)$-to-$\left(1-\rho_{1}\right)$ for the complement event $Q^{\prime }\left(S\right)$. In the original definition, the constraint for Pr[Q(S)|Y=y] includes equality. Here, we remove the equality constraint for consistency with the context. They proved that in the ε-LDP scenario, the sufficient condition to prevent both upward $\rho_{1}$-to-$\rho_{2}$ and downward $\rho_{2}$-to-$\rho_{1}$ privacy breach is

(A1) $$e^{\varepsilon }\leq \frac{\rho_{2}}{\rho_{1}}\cdot \frac{1-\rho_{1}}{1-\rho_{2}}.$$

Here, we provide sufficient conditions to prevent upward $\rho_{1}$-to-$\rho_{2}$ and downward $\rho_{2}$-to-$\rho_{1}$ in terms of min- and max-lift as follows:

**Proposition** **A1.**
*To prevent upward and downward privacy breaches it is sufficient to have:*

$$\frac{1-\rho_{2}}{1-\rho_{1}}\leq \Psi \left(y\right),\;\Lambda \left(y\right)\leq \frac{\rho_{2}}{\rho_{1}},\;\forall y\in Y.$$

**Proof.** We prove this for an upward privacy breach on a property Q(S) and a downward privacy breach on its complement ¬Q(S). For any property Q(S) with $\mathrm{Pr}\left[Q\left(S\right)\right]\leq \rho_{1}$ we have

$$\begin{array}{c} \mathrm{Pr}\left[Q\left(S\right)|Y=y\right]=\sum_{s\in Q(S)}P_{S|Y}\left(s|y\right)=\sum_{s\in Q(S)}\frac{P_{S}\left(s\right).P_{Y|S}\left(y|s\right)}{P_{Y}\left(y\right)}\leq \sum_{s\in Q(S)}P_{S}\left(s\right)\max_{s}\frac{P_{Y|S}\left(y|s\right)}{P_{Y}\left(y\right)}=\Lambda \left(y\right)\mathrm{Pr}\left[Q\left(S\right)\right]. \end{array}$$

Since $\mathrm{Pr}\left[Q\left(S\right)\right]\leq \rho_{1}$, if $\Lambda \left(y\right)\leq \frac{\rho_{2}}{\rho_{1}}$, then $\mathrm{Pr}\left[Q\left(S\right)|Y=y\right]\leq \rho_{2}$ and there is no upward privacy breach for Q(S). Similarly, for downward privacy breach on ¬Q(S), we have

$$\begin{array}{cc} \mathrm{Pr}[\neg Q(S)|Y=y] & =\sum_{s\in \neg Q(S)}P_{S|Y}\left(s|y\right)=\sum_{s\in \neg Q(S)}\frac{P_{S}\left(s\right).P_{Y|S}\left(y|s\right)}{P_{Y}\left(y\right)}\\ \; & \geq \sum_{s\in \neg Q(S)}P_{S}\left(S\right)\min_{s}\frac{P_{Y|S}\left(y|s\right)}{P_{Y}\left(y\right)}=\Psi \left(y\right)\mathrm{Pr}\left[\neg Q\left(S\right)\right]. \end{array}$$

Since $\mathrm{Pr}\left[\neg Q\left(S\right)\right]\geq 1-\rho_{1}$, if $\Psi \left(y\right)\geq \frac{1-\rho_{2}}{1-\rho_{1}}$, then $\mathrm{Pr}\left[\neg Q\left(S\right)|Y=y\right]\geq 1-\rho_{2}$ and there is no downward privacy breach for ¬Q(S). □

## Appendix B

1. For MI, we have
  $$I\left(S;Y\right)=E_{P_{SY}}\left[i\left(S,Y\right)\right]\leq E_{P_{SY}}\left[\varepsilon_{u}\right]=\varepsilon_{u}.$$
2. - For the total variation distance, we have
    $$\begin{array}{cc} T(S;Y)\; & =\frac{1}{2}\sum_{y\in Y}P_{Y}\left(y\right)\sum_{s\in S}P_{S}\left(s\right)|l\left(s,y\right)-1|\leq \frac{1}{2}\sum_{y\in Y}P_{Y}\left(y\right)\sum_{s\in S}P_{S}\left(s\right)\left|\Lambda \left(y\right)-1\right|\\ \; & =\frac{1}{2}\sum_{y\in Y}P_{Y}\left(y\right)|\Lambda \left(y\right)-1|\leq \frac{1}{2}\sum_{y\in Y}P_{Y}\left(y\right)\left|e^{\varepsilon_{u}}-1\right|=\frac{1}{2}\left(e^{\varepsilon_{u}}-1\right). \end{array}$$
  - For $\chi^{2}$-divergence, we have
    $$\begin{array}{cc} \chi^{2}\left(S;Y\right) & =\sum_{y\in Y}P_{Y}\left(y\right)\sum_{s\in S}P_{S}\left(s\right){\left(l(s,y)-1\right)}^{2}\leq \sum_{y\in Y}P_{Y}\left(y\right)\sum_{s\in S}P_{S}\left(s\right){\left(\Lambda (y)-1\right)}^{2}\\ \; & =\sum_{y\in Y}P_{Y}\left(y\right){\left(\Lambda (y)-1\right)}^{2}\leq \sum_{y\in Y}P_{Y}\left(y\right){\left(e^{\varepsilon_{u}}-1\right)}^{2}={\left(e^{\varepsilon_{u}}-1\right)}^{2}. \end{array}$$
3. - For Sibson MI, we have
    $$\begin{array}{cc} I_{\alpha }^{S}\left(S;Y\right)\; & =\frac{\alpha }{\alpha -1}\log\sum_{y\in Y}P_{Y}\left(y\right){\left(\sum_{s\in S}P_{S}\left(s\right)l{\left(s,y\right)}^{\alpha }\right)}^{1/\alpha }\\ \; & \leq \frac{\alpha }{\alpha -1}\log\sum_{y\in Y}P_{Y}\left(y\right){\left(\sum_{s\in S}P_{S}\left(s\right)\Lambda {\left(y\right)}^{\alpha }\right)}^{1/\alpha }\\ \; & \leq \frac{\alpha }{\alpha -1}\log\sum_{y\in Y}P_{Y}\left(y\right){\left(\sum_{s\in S}P_{S}\left(s\right)e^{\varepsilon_{u}\alpha }\right)}^{1/\alpha }=\frac{\varepsilon_{u}\alpha }{\alpha -1}. \end{array}$$
  - For Arimoto MI, we have
    $$\begin{array}{cc} I_{\alpha }^{A}\left(S;Y\right)\; & =\frac{\alpha }{\alpha -1}\log\sum_{y\in Y}P_{Y}\left(y\right){\left(\sum_{s\in S}P_{S_{\alpha }}\left(s\right)l{\left(s,y\right)}^{\alpha }\right)}^{1/\alpha }\\ \; & \leq \frac{\alpha }{\alpha -1}\log\sum_{y\in Y}P_{Y}\left(y\right){\left(\sum_{s\in S}P_{S_{\alpha }}\left(s\right)\Lambda {\left(y\right)}^{\alpha }\right)}^{1/\alpha }\\ \; & \leq \frac{\alpha }{\alpha -1}\log\sum_{y\in Y}P_{Y}\left(y\right){\left(\sum_{s\in S}P_{S_{\alpha }}\left(s\right)e^{\varepsilon_{u}\alpha }\right)}^{1/\alpha }=\frac{\varepsilon_{u}\alpha }{\alpha -1}, \end{array}$$
    where $P_{S_{\alpha }}\left(s\right)=\frac{P_{S}{\left(s\right)}^{\alpha }}{\sum_{s\in S}P_{S}{\left(s\right)}^{\alpha }}.$
4. In LDP, for all y∈Y, we have
  $$\begin{array}{c} \Gamma \left(y\right)=\underset{s,s^{\prime }\in S}{\sup}\frac{P_{Y|S}\left(y|s\right)}{P_{Y|S}\left(y|s^{\prime }\right)}=\frac{\max_{s\in S}P_{Y|S}\left(y|s\right)}{\min_{s\in S}P_{Y|S}\left(y|s\right)}=\frac{\max_{s\in S}P_{Y|S}\left(y|s\right)/P_{Y}\left(y\right)}{\min_{s\in S}P_{Y|S}\left(y|s\right)/P_{Y}\left(y\right)}=\frac{\Lambda (y)}{\Psi (y)}\leq \frac{e^{\varepsilon_{u}}}{e^{-\varepsilon_{l}}}=e^{\varepsilon_{l}+\varepsilon_{u}}. \end{array}$$

## Appendix C

Here, we prove that *X*-invariant randomisation minimises privacy leakage in $X_{H}$ for LDP.

**Proposition** **A2.**
*A randomisation r(y|x), $x,y,\in X_{H}$ can attain $\left(\varepsilon ,X_{H}\right)$-LDP if and only if:*

(A2)
$$\Gamma_{LDP}\left(X_{H}\right)=\frac{\max_{s\in S}P\left(X_{H}|s\right)}{\min_{s\in S}P\left(X_{H}|s\right)}\leq e^{\varepsilon }.$$

**Proof.** Sufficient condition: Consider an X-invariant randomization where r(y|x)=R(y), $\forall x\in X_{H}$, and $y\in Y_{H}$. If (A2) holds, then for all $s,s^{\prime }\in S$, we have

$$\begin{array}{cc} \frac{P(X_{H}|s)}{P(X_{H}|s^{\prime })} & \leq \frac{\max_{s\in S}P\left(X_{H}|s\right)}{\min_{s\in S}P\left(X_{H}|s\right)}\leq e^{\varepsilon }\Rightarrow \\ P(X_{H}|s) & \leq P\left(X_{H}|s^{\prime }\right)e^{\varepsilon }\Rightarrow \\ R\left(y\right)P\left(X_{H}|s\right) & \leq R\left(y\right)P\left(X_{H}|s^{\prime }\right)e^{\varepsilon }\Rightarrow \\ \sum_{x\in X_{H}}r\left(y|x\right)P_{X|S}\left(x|s\right) & \leq \sum_{x\in X_{H}}r\left(y|x\right)P_{X|S}\left(x|s^{\prime }\right)e^{\varepsilon }\Rightarrow \\ P_{Y|S}\left(y|s\right) & \leq P_{Y|S}\left(y|s^{\prime }\right)e^{\varepsilon }. \end{array}$$

For the necessary condition, note that for all $s,s^{\prime }\in S$ and $y\in Y_{H}$, we have

$$\begin{array}{c} P_{Y|S}\left(y|s\right)\leq e^{\varepsilon }P_{Y|S}\left(y|s^{\prime }\right)\Rightarrow \sum_{x\in X_{H}}r\left(y|x\right)P_{X|S}\left(x|s\right)\leq e^{\varepsilon }\sum_{x\in X_{H}}r\left(y|x\right)P_{X|S}\left(x|s^{\prime }\right), \end{array}$$

then by a summation over all $y\in Y_{H}$ on both sides, we obtain

(A3) $$\begin{array}{cc} & \sum_{x\in X_{H}}P_{X|S}\left(x|s\right)\underset{=1}{\underset{︸}{\sum_{y\in Y_{H}}r\left(y|x\right)}}\leq e^{\varepsilon }\sum_{x\in X_{H}}P_{X|S}\left(x|s^{\prime }\right)\underset{=1}{\underset{︸}{\sum_{y\in Y_{H}}r\left(y|x\right)}}\\ \; & \Rightarrow P\left(X_{H}|s\right)\leq P\left(X_{H}|s^{\prime }\right)e^{\varepsilon }\Rightarrow \frac{P(X_{H}|s)}{P(X_{H}|s^{\prime })}\leq e^{\varepsilon }. \end{array}$$

Because (A3) holds for all $s,s^{\prime }\in S$, we have

$$\max_{s,s^{\prime }\in S}\frac{P(X_{H}|s)}{P(X_{H}|s^{\prime })}=\frac{\max_{s\in S}P\left(X_{H}|s\right)}{\min_{s\in S}P\left(X_{H}|s\right)}\leq e^{\varepsilon }.$$

□

## Appendix D

1. For $\ell_{1}$-lift, we have
  $$\begin{array}{c} \Lambda_{\ell_{1}}\left(y\right)=\sum_{s\in S}P_{S}\left(s\right)|l\left(s,y\right)-1|\leq \sum_{s\in S}P_{S}\left(s\right)\left|\Lambda \left(y\right)-1\right|=\Lambda \left(y\right)-1\leq e^{\varepsilon_{u}}-1. \end{array}$$
2. For $\chi^{2}$-lift, we have
  $$\begin{array}{c} \Lambda_{\chi^{2}}\left(y\right)=\sum_{s\in S}P_{S}\left(s\right){\left(l(s,y)-1\right)}^{2}\leq \sum_{s\in S}P_{S}\left(s\right){\left(\Lambda (y)-1\right)}^{2}={\left(\Lambda (y)-1\right)}^{2}\leq {\left(e^{\varepsilon_{u}}-1\right)}^{2}. \end{array}$$
3. For the α-lift, we have
  $$\begin{array}{c} \Lambda_{\alpha }^{S}\left(y\right)={\left(\sum_{s\in S}P_{S}\left(s\right)l{\left(s,y\right)}^{\alpha }\right)}^{1/\alpha }\leq {\left(\sum_{s\in S}P_{S}\left(s\right)\Lambda {\left(y\right)}^{\alpha }\right)}^{1/\alpha }=\Lambda \left(y\right)\leq e^{\varepsilon_{u}}. \end{array}$$

## Appendix E

If $s_{y}=\arg\;\max_{s\in S}l\left(s,y\right)$, then we have $\Lambda \left(y\right)=l\left(s_{y},y\right)$. Recall that $\bar{y}=\arg\;\max_{y\in Y}\left[\Lambda \left(y\right)\right]$.

1. When $\max_{y\in Y}\Lambda_{\ell_{1}}\left(y\right)\leq \varepsilon$, for all y∈Y, we have
  $$\begin{array}{cc} \Lambda_{\ell_{1}}\left(y\right) & =\sum_{s\in S}P_{S}\left(s\right)|l\left(s,y\right)-1|\leq \varepsilon \Rightarrow P_{S}\left(s_{y}\right)\left|l\left(s_{y},y\right)-1\right|=P_{S}\left(s_{y}\right)\left(\Lambda \left(y\right)-1\right)\leq \varepsilon , \end{array}$$
  which results in
  $$\max_{y\in Y}\Lambda \left(y\right)\leq \frac{\varepsilon }{P_{S}\left(s_{\bar{y}}\right)}+1.$$
2. When $\max_{y\in Y}\Lambda_{\chi^{2}}\left(y\right)\leq \varepsilon$, for all y∈Y, we have
  $$\begin{array}{cc} \Lambda_{\chi^{2}}\left(y\right) & =\sum_{s\in S}P_{S}\left(s\right){\left(l(s,y)-1\right)}^{2}\leq \varepsilon \Rightarrow P_{S}\left(s_{y}\right){\left(l(s_{y},y)-1\right)}^{2}=P_{S}\left(s_{y}\right){\left(\Lambda \left(y\right)-1\right)}^{2}\leq \varepsilon , \end{array}$$
  which results in
  $$\max_{y\in Y}\Lambda \left(y\right)\leq \sqrt{\frac{\varepsilon }{P_{S}\left(s_{\bar{y}}\right)}}+1.$$
3. When $\max_{y\in Y}\Lambda_{\alpha }^{S}\left(y\right)\leq \varepsilon$, for all y∈Y, we have
  $$\begin{array}{cc} \Lambda_{\alpha }^{S}\left(y\right) & ={\left(\sum_{s\in S}P_{S}\left(s\right)l{\left(s,y\right)}^{\alpha }\right)}^{1/\alpha }\leq \varepsilon \Rightarrow P_{S}\left(s_{y}\right)l{\left(s_{y},y\right)}^{\alpha }=P_{S}\left(s_{y}\right)\Lambda {\left(y\right)}^{\alpha }\leq \varepsilon^{\alpha }, \end{array}$$
  which results in
  $$\max_{y\in Y}\Lambda \left(y\right)\leq \frac{\varepsilon }{P_{S}{\left(s_{\bar{y}}\right)}^{\frac{1}{\alpha }}}.$$

## Appendix F

Since $\left(\varepsilon_{l},\varepsilon_{u}\right)$-ALIP is satisfied, for all y∈Y, we have $e^{-\varepsilon_{u}}\leq \frac{1}{l(s,y)}\leq e^{\varepsilon_{l}}$ and $\max_{s}\left(\frac{1}{l(s,y)}\right)=\frac{1}{\Psi (y)}$.

1. For $\ell_{1}$-lift-inverse, we have
  $$\begin{array}{c} \Psi_{\ell_{1}}\left(y\right)=\sum_{s\in S}P_{S}\left(s\right)\left|\frac{1}{l(s,y)}-1\right|\leq \sum_{s\in S}P_{S}\left(s\right)\left|\frac{1}{\Psi (y)}-1\right|=\frac{1}{\Psi (y)}-1\leq e^{\varepsilon_{l}}-1. \end{array}$$
2. For $\chi^{2}$-lift-inverse, we have
  $$\begin{array}{cc} \Psi_{\chi^{2}}\left(y\right) & =\sum_{s\in S}P_{S}\left(s\right){\left(\frac{1}{l(s,y)}-1\right)}^{2}\leq \sum_{s\in S}P_{S}\left(s\right){\left(\frac{1}{\Psi (y)}-1\right)}^{2}={\left(\frac{1}{\Psi (y)}-1\right)}^{2}\leq {\left(e^{\varepsilon_{l}}-1\right)}^{2}. \end{array}$$
3. For α-lift-inverse, we have
  $$\begin{array}{c} \Psi_{\alpha }^{S}\left(y\right)={\left(\sum_{s\in S}P_{S}\left(s\right){\left(\frac{1}{l(s,y)}\right)}^{\alpha }\right)}^{\frac{1}{\alpha }}\leq {\left(\sum_{s\in S}P_{S}\left(s\right){\left(\frac{1}{\Psi (y)}\right)}^{\alpha }\right)}^{\frac{1}{\alpha }}=\frac{1}{\Psi (y)}\leq e^{\varepsilon_{l}}. \end{array}$$

## Appendix G

If $s_{y}=\arg\;\min_{s}l\left(s,y\right)$, then we have $\Psi \left(y\right)=l\left(s_{y},y\right)$. Recall that $\underline{y}=\arg\;\min_{y}\left[\Psi \left(y\right)\right].$

1. When $\max_{y\in Y}\Psi_{\ell_{1}}\left(y\right)\leq \varepsilon$, for all y∈Y, we have
  $$\begin{array}{cc} \Psi_{\ell_{1}}\left(y\right) & =\sum_{s\in S}P_{S}\left(s\right)\left|\frac{1}{l(s,y)}-1\right|\leq \varepsilon \Rightarrow P_{S}\left(s_{y}\right)\left|\frac{1}{l(s_{y},y)}-1\right|=P_{S}\left(s_{y}\right)\left(\frac{1}{\Psi (y)}-1\right)\leq \varepsilon , \end{array}$$
  which results in
  $$\min_{y\in Y}\Psi \left(y\right)\geq \frac{P_{S}\left(s_{\underline{y}}\right)}{\varepsilon +P_{S}\left(s_{\underline{y}}\right)}.$$
2. When $\max_{y\in Y}\Psi_{\chi^{2}}\left(y\right)\leq \varepsilon$, for all y∈Y, we have
  $$\begin{array}{cc} \Psi_{\chi^{2}}\left(y\right) & =\sum_{s\in S}P_{S}\left(s\right){\left(\frac{1}{l(s,y)}-1\right)}^{2}\leq \varepsilon \Rightarrow P_{S}\left(s_{y}\right){\left(\frac{1}{l(s_{y},y)}-1\right)}^{2}=P_{S}\left(s_{y}\right){\left(\frac{1}{\Psi (y)}-1\right)}^{2}\leq \varepsilon , \end{array}$$
  which results in
  $$\min_{y\in Y}\Psi \left(y\right)\geq \frac{\sqrt{P_{S}\left(s_{\underline{y}}\right)}}{\sqrt{\varepsilon }+\sqrt{P_{S}\left(s_{\underline{y}}\right)}}.$$
3. When $\max_{y\in Y}\Psi_{\alpha }^{S}\left(y\right)\leq \varepsilon$, for all y∈Y, we have
  $$\begin{array}{cc} \Psi_{\alpha }^{S}\left(y\right) & ={\left(\sum_{s\in S}P_{S}\left(s\right){\left(\frac{1}{l(s,y)}\right)}^{\alpha }\right)}^{1/\alpha }\leq \varepsilon \Rightarrow P_{S}\left(s_{y}\right){\left(\frac{1}{l(s_{y},y)}\right)}^{\alpha }=P_{S}\left(s_{y}\right){\left(\frac{1}{\Psi (y)}\right)}^{\alpha }\leq \varepsilon^{\alpha }, \end{array}$$
  which results in
  $$\min_{y\in Y}\Psi \left(y\right)\geq \varepsilon^{-1}P_{S}{\left(s_{\underline{y}}\right)}^{\frac{1}{\alpha }}.$$

## References

[1] Dwork C., McSherry F., Nissim K., Smith A., Halevi S., Rabin T. (2006). Calibrating Noise to Sensitivity in Private Data Analysis. Theory of Cryptography.

[2] Dwork C. Differential Privacy. Proceedings of the 33rd International Colloquium on Automata, Languages and Programming, part II (ICALP 2006).

[3] Dwork C. (2011). Differential privacy. Encyclopedia of Cryptography and Security.

[4] Dwork C., Roth A. (2014). The algorithmic foundations of differential privacy. Found. Trends Theor. Comput. Sci..

[5] Kasiviswanathan S.P., Lee H.K., Nissim K., Raskhodnikova S., Smith A. (2011). What can we learn privately?. SIAM J. Comput..

[6] Duchi J.C., Jordan M.I., Wainwright M.J. Local Privacy and Statistical Minimax Rates. Proceedings of the 2013 IEEE 54th Annual Symposium on Foundations of Computer Science.

[7] Kairouz P., Oh S., Viswanath P. (2014). Extremal mechanisms for local differential privacy. Adv. Neural Inf. Process. Syst..

[8] Sarwate A.D., Sankar L. A rate-disortion perspective on local differential privacy. Proceedings of the 2014 52nd Annual Allerton Conference on Communication, Control, and Computing (Allerton).

[9] Kalantari K., Sankar L., Sarwate A.D. (2018). Robust Privacy-Utility Tradeoffs Under Differential Privacy and Hamming Distortion. IEEE Trans. Inf. Forensics Secur..

[10] Sankar L., Rajagopalan S.R., Poor H.V. (2013). Utility-privacy tradeoffs in databases: An information-theoretic approach. IEEE Trans. Inf. Forensics Secur..

[11] Jiang B., Li M., Tandon R. Context-aware Data Aggregation with Localized Information Privacy. Proceedings of the 2018 IEEE Conference on Communications and Network Security (CNS).

[12] Makhdoumi A., Salamatian S., Fawaz N., Médard M. From the Information Bottleneck to the Privacy Funnel. Proceedings of the 2014 IEEE Information Theory Workshop (ITW 2014).

[13] Salamatian S., du Pin Calmon F., Fawaz N., Makhdoumi A., Médard M. (2020). Privacy-Utility Tradeoff and Privacy Funnel. http://www.mit.edu/~salmansa/files/privacy_TIFS.pdf.

[14] Issa I., Kamath S., Wagner A.B. An operational measure of information leakage. Proceedings of the 2016 Annual Conference on Information Science and Systems (CISS).

[15] Issa I., Kamath S., Wagner A.B. Maximal leakage minimization for the Shannon cipher system. Proceedings of the 2016 IEEE International Symposium on Information Theory (ISIT).

[16] Issa I., Wagner A.B., Kamath S. (2020). An Operational Approach to Information Leakage. IEEE Trans. Inf. Theory.

[17] Liao J., Kosut O., Sankar L., Calmon F.P. A tunable measure for information leakage. Proceedings of the IEEE International Symposium on Information Theory (ISIT).

[18] Du Pin Calmon F., Fawaz N. Privacy against statistical inference. Proceedings of the 50th Annual Allerton Conference on Communication, Control, and Computing (Allerton).

[19] Jiang B., Li M., Tandon R. Local Information Privacy with Bounded Prior. Proceedings of the 2019 IEEE International Conference on Communications (ICC).

[20] Seif M., Tandon R., Li M. Context Aware Laplacian Mechanism for Local Information Privacy. Proceedings of the 2019 IEEE Information Theory Workshop (ITW).

[21] Jiang B., Li M., Tandon R. (2020). Local Information Privacy and Its Application to Privacy-Preserving Data Aggregation. IEEE Trans. Dependable Secur. Comput..

[22] Jiang B., Seif M., Tandon R., Li M. (2021). Context-Aware Local Information Privacy. IEEE Trans. Inf. Forensics Secur..

[23] Ding N., Liu Y., Farokhi F. A Linear Reduction Method for Local Differential Privacy and Log-lift. Proceedings of the 2021 IEEE International Symposium on Information Theory (ISIT).

[24] Hsu H., Asoodeh S., Calmon F.P. Information-Theoretic Privacy Watchdogs. Proceedings of the 2019 IEEE International Symposium on Information Theory (ISIT).

[25] Sadeghi P., Ding N., Rakotoarivelo T. On Properties and Optimization of Information-theoretic Privacy Watchdog. Proceedings of the 2020 IEEE Information Theory Workshop (ITW).

[26] Zarrabian M.A., Ding N., Sadeghi P., Rakotoarivelo T. Enhancing utility in the watchdog privacy mechanism. Proceedings of the ICASSP 2022—2022 IEEE International Conference on Acoustics, Speech and Signal Processing (ICASSP).

[27] Razeghi B., Calmon F., Gunduz D., Voloshynovskiy S. (2020). On Perfect Obfuscation: Local Information Geometry Analysis. arXiv.

[28] Lopuhaä-Zwakenberg M., Tong H., Škorić B. (2021). Data Sanitisation Protocols for the Privacy Funnel with Differential Privacy Guarantees. Int. J. Adv. Secur..

[29] Evfimievski A., Gehrke J., Srikant R. Limiting Privacy Breaches in Privacy Preserving Data Mining. Proceedings of the Twenty-Second ACM SIGMOD-SIGACT-SIGART Symposium on Principles of Database Systems, PODS ’03.

[30] Saeidian S., Cervia G., Oechtering T.J., Skoglund M. (2022). Pointwise Maximal Leakage. arXiv.

[31] Fernandes N., McIver A., Sadeghi P. (2022). Explaining epsilon in differential privacy through the lens of information theory. arXiv.

[32] Zamani A., Oechtering T.J., Skoglund M. (2022). Data Disclosure With Non-Zero Leakage and Non-Invertible Leakage Matrix. IEEE Trans. Inf. Forensics Secur..

[33] Zamani A., Oechtering T.J., Skoglund M. (2021). A Design Framework for Strongly *χ*^2^-Private Data Disclosure. IEEE Trans. Inf. Forensics Secur..

[34] Ding N., Zarrabian M.A., Sadeghi P. *α*-Information-theoretic Privacy Watchdog and Optimal Privatization Scheme. Proceedings of the 2021 IEEE International Symposium on Information Theory (ISIT).

[35] Rassouli B., Gunduz D. (2019). Optimal utility-privacy trade-off with total variation distance as a privacy measure. IEEE Trans. Inf. Forensics Secur..

[36] Asoodeh S., Diaz M., Alajaji F., Linder T. (2018). Estimation efficiency under privacy constraints. IEEE Trans. Inf. Theory.

[37] Rassouli B., Rosas F.E., Gunduz D. (2019). Data Disclosure under Perfect Sample Privacy. arXiv.

[38] Becker B., Kohavi R. Adult. UC Irvine Machine Learning Repository. https://archive-beta.ics.uci.edu/dataset/2/adult.

[39] Liu Y., Sadeghi P., Arbabjolfaei F., Kim Y.H. (2020). Capacity Theorems for Distributed Index Coding. IEEE Trans. Inf. Theory.

[40] Wang H., Vo L., Calmon F.P., Médard M., Duffy K.R., Varia M. (2019). Privacy With Estimation Guarantees. IEEE Trans. Inf. Theory.

